# Biocatalytic reductive amination with CRISPR-Cas9 engineered yeast

**DOI:** 10.1038/s41598-025-01182-0

**Published:** 2025-05-15

**Authors:** Arne Hagman, Olof Stenström, Göran Carlström, Mikael Akke, Carl Grey, Magnus Carlquist

**Affiliations:** 1 Division of Biotechnology and Applied Microbiology, Lund University, Lund, Sweden; 2 Division of Biophysical Chemistry, Center for Molecular Protein Science, Lund University, Lund, Sweden

**Keywords:** Biocatalysis, Transferases, Genetic engineering, CRISPR-Cas9 genome editing, Saccharomyces cerevisiae, Metabolic engineering, Fungal physiology

## Abstract

Metabolically engineered baker’s yeast can be used to produce chiral amines through whole-cell bioconversion of prochiral ketones. This study investigates the modulation of the alanine-pyruvate metabolic node to enhance reductive amination, using the stereoselective conversion of benzylacetone to (*S*)-1-methyl-3-phenylpropylamine (MPPA) as a model reaction. Chromosomal integration of multiple copies of the promiscuous omega transaminase from *Chromobacterium violaceum* (cv-ATA) resulted in an active yeast catalyst. Physiological characterization in bioreactors under aerobic batch cultivation revealed that amine production occurred only under post-diauxic growth on ethanol. To reduce native alanine utilization, the endogenous alanine aminotransferase (ALT1) was knocked out and replaced with cv-ATA. To rapidly employ this strategy in other strains, a simple CRISPR/cas9 method for universal gene replacement was developed. The replacement of ALT1 with cv-ATA improved the reaction by 2.6-fold compared to the control strain with intact ALT1. NMR measurements of metabolites originating from ^15^N L-alanine and ^13^C glucose indicated that pyruvate formation during growth on glucose inhibited amine production. Under optimal conditions, the biocatalytic bioconversion of benzylacetone to MPPA reached a yield of 58%.

**Supplementary Information:**

The online version contains supplementary material available at 10.1038/s41598-025-01182-0.

## Introduction

*Saccharomyces cerevisiae* plays an important role in industrial biotechnology and has been successfully engineered for production of various commodity chemicals, biofuels and bioactive compounds^1,2^. Relatively little attention has however been given to the development of yeast for production of amines, despite their central position as building blocks for pharmaceuticals (e.g., analgesics, antidiabetic, and antihypertensive drugs), agrochemicals (including herbicides, fungicides, and insecticides), solvents, vulcanization accelerators for rubber and plasticizers, and polymers (e.g., polyamides)^3,4^. Currently, amines are primarily synthesized by dehydrating suitable alcohols with ammonia using a catalyst under harsh conditions. The use of enzyme or microbial catalysts has several potential benefits over chemical methods, such as high regio- and stereo-selectivity, operating under ambient conditions, generating less nitrogenous waste, and enabling significant process intensification by supplying enzymes and reactants in situ^5^.

We have previously developed *S. cerevisiae* strains over-expressing specific amine transaminases (ATAs) (EC 2.6.1.1) of plant or bacterial origin for kinetic resolution of racemic amines with glucose as source of amine acceptor^6–9^. In a previous comparative study between *S. cerevisiae* and *E. coli* over-expressing recombinant ATA for whole-cell amine production, the yeast displayed a higher robustness to process conditions enabling bioconversion over a longer time period^8^. Furthermore, *S. cerevisiae* was shown to have high capacity to supply the prosthetic group pyrodoxal-5-phosphate (PLP) *de* novo^6^. This is an advantage over in vitro enzyme catalysis or whole-cell biocatalysis with the more commonly used *Escherichia coli*, where PLP is typically added to the reaction broth^10^.

ATA-based reductive amination of carbonyl compounds, i.e., the reverse reaction compared to kinetic resolution, on the other hand, has shown to be challenging in yeast due to difficulty to achieve a favorable reaction equilibrium towards the amine product^11,12^. Previously, the methylotrophic yeast *Pichia pastoris* expressing a recombinant ATA was developed for reductive amination of the prochiral ketone benzylacetone (BA) to (*S*)−1-methyl-3-phenylpropylamine (MPPA)^13^. To push the reaction to the target product, the whole-cell bioconversion was operated at a high-cell density of *P. pastoris*, and the high-value compound (*S*)−1-phenylethylamine ((*S*)−1-PEA)) was used as amine donor. Chiral amine production with endogenous amine donors, which could be supplied in vivo by the yeast would be an attractive alternative, but achieving high specific productivity has hitherto not been described. Therefore, methods that modulate the intracellular environment of yeast in favor of the amine product need to be developed before this can be realized.

*S. cerevisiae* has previously been shown to be excellent expression host for the transaminase Cv-ATA^6^. Cv-ATA displays high activity in vivo for the conversion of amines to ketones with pyruvate as amine acceptor^6^, and high activity ex vivo in crude cell extract for the conversion of ketones to amines with *L*-alanine as amine donor. However, in vivo reductive amination has previously been shown to be difficult despite adding excess amount of *L*-alanine in the reaction broth^6^. *S. cerevisiae* is a Crabtree positive yeast that rapidly converts glucose to ethanol, via pyruvate under both fermentative and respiratory conditions^14,15^. *L*-alanine is efficiently converted to pyruvate by alanine aminotransferase, encoded by ALT1^16^, which may compete with heterologous transaminase reactions. In this study, we investigated the effects of engineering the alanine-pyruvate metabolic node on cell growth and bio-catalytic amine production. Whole-cell bioconversion of BA to MPPA by recombinant ATA from *C. violaceum* (Cv-ATA) was used as model. A CRISPR/Cas9-method for universal gene replacement was developed and applied to replace ALT1 with Cv-ATA under constitutive regulation. Constructed strains were characterized in bioreactors, revealing conditions that favored reductive amination. HPLC profiling of metabolites, and NMR monitoring of reactants with ^15^N *L*-alanine as amine donor and ^13^C glucose were performed and provided a molecular explanation for the observations.

## Results and discussion

### Screening for amine-donor and whole-cell bioconversion

**Fig. 1:**
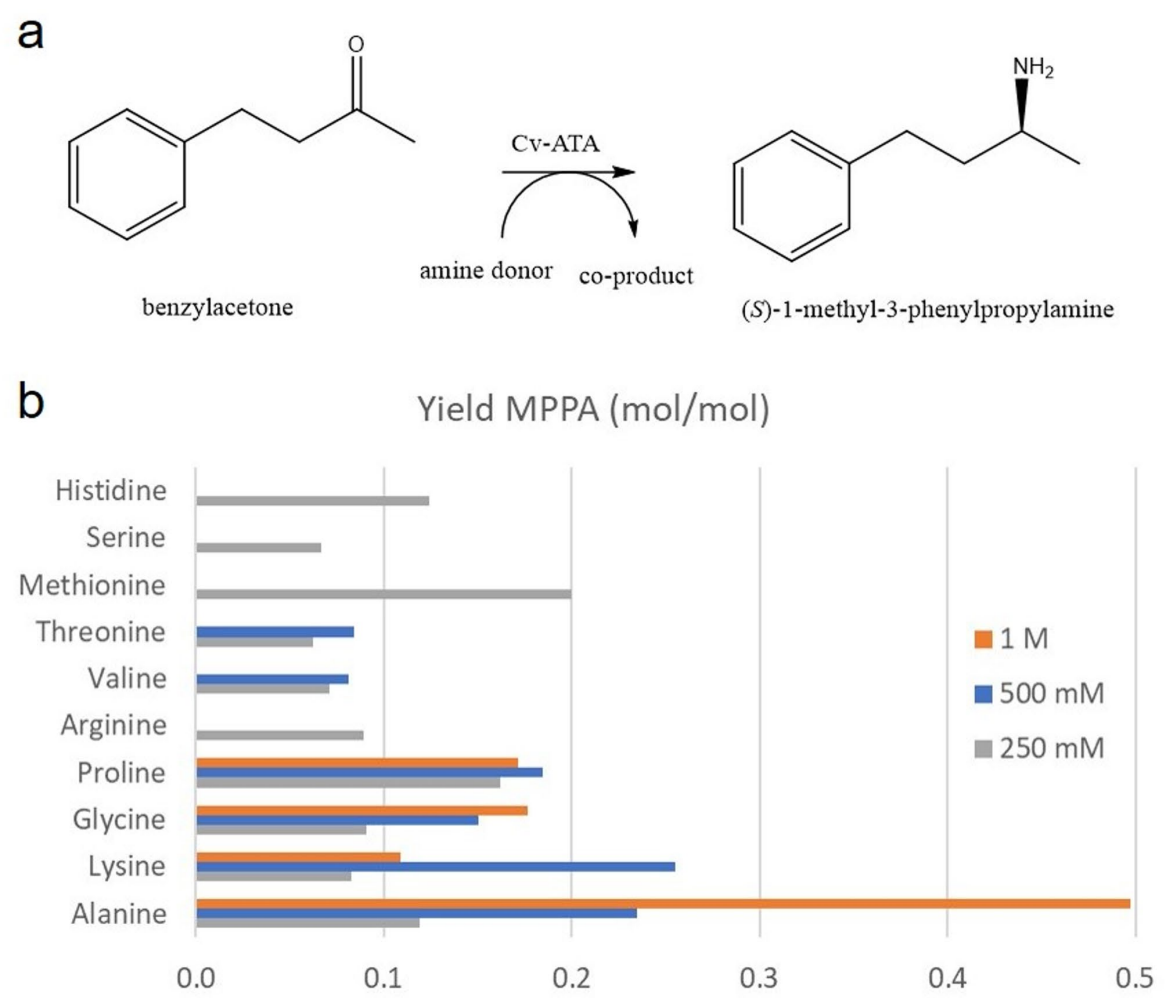
(A) Yeast expressing ATA (TMB4375) converts the pro-chiral ketone benzylacetone (BA) to the corresponding chiral-amine (*S*)−1-methyl-3-phenylpropylamine (MPPA). ATA catalyzes the transfer of an amine from the amine donor, which is converted to the corresponding ketone co-product. (B) Ten amino acids were screened for their potency as amine donor for this reaction in yeast. Depending on their solubility, amino acids were tested up to 1 M (Ala, Lys, Gly, Pro, Arg), 500 mM (Val, Thr), or 250 mM (Met, Ser, His).

A range of amino acids may potentially be used as amine donor for the production of chiral amines in yeast that express recombinant aminotransaminase (ATA from *C. violaceum*) (Table 1, S1). MPPA production depends on the availability of endogenous amine donors that are accepted by recombinant ATA. The yeast TMB4375^6^ contain six copies of ATA, and it was used to screen for amine donors with the highest yields of MPPA (Fig. 1). TMB4131 was the reference strain in this study, and it does not contain ATA. We could not detect any MPPA produced by the reference strain at any concentrations of amine donors tested.

**Table 1:** Engineered yeast strains and their origin.

Strain	Reference	Background	Growth profile	Genotype* rpl22p-mEGFP	ALT1	ATA
cen.pk113-7D		wt	I	-	1	-
TMB4131	Weber^6^	cen.pk2-1 C	ND	-	1	-
TMB4375	Weber^6^	cen.pk2-1 C	ND	-	1	6
TMBAH13	This study	cen.pk113-7D	ND	1	1	-
TMBAH19	This study	cen.pk2-1 C	I	1	1	-
TMBAH25	This study	cen.pk2-1 C	III	1	1	6
TMBAH57	This study	cen.pk113-7D	ND	1	-	-
TMBAH58	This study	cen.pk2-1 C	II	1	-	-
TMBAH59	This study	cen.pk2-1 C	III	1	-	6
TMBAH60	This study	cen.pk113-7D	III	1	-	1
TMBAH61	This study	cen.pk2-1 C	II	1	-	1
TMBAH62	This study	cen.pk2-1 C	III	1	-	7

*see supplementary table S1 for more detailed information on genotype.

Not determined (ND).

Data availability.

DNA sequences in this study are available in the Supplementary Information. Other relevant datasets used and/or analyzed during the study can be made available upon reasonable request to the corresponding author.

Code availability.

The custom made Matlab script that was used to analyze all flowcytometry data in this study can be downloaded from the following GitHub repository: https://github.com/MicrobialEngineeringGroupTMB/.

We utilized a simple explorative screening approach that did not take into account any possible differences in uptake kinetics nor any potential toxicity of amino acids in high amounts. The purpose of the screening was to identify good candidates that could be used by yeast for chiral amine production. We screened several amino acids with sufficient solubility in water at 250 mM up to 1 M, for conversion of benzylacetone to MPPA (Fig. 1a, Supplementary Table S2). Of all twenty proteinogenic amino acids, only five with the highest solubility could be tested up to 1 M; these comprise alanine, lysine, glycine, proline, and arginine. Two amino acids, valine and threonine, could be tested up to 500 mM. And three amino acids, methionine, serine, and histidine could be tested only at 250 mM. The remaining ten amino acids had too low solubility in aqueous solution to be considered as good candidates. The starting pH was in the range 6–7, and the end pH was in the range 5–6 for all amino acids except for arginine. At concentrations above 250 mM, the pH deviated higher than 10 for arginine, and these results were for that reason not included in the analysis. The amino acid that consistently performed best, at all concentrations throughout the 6 days that the experiment lasted, was alanine (Fig. 1b, Supplementary Table S2). The highest detected yields of MPPA were close to 50% at 1 M, and 25% at 250 mM alanine. Other amino acids that performed well were lysine, glycine, proline and methionine. However, no MPPA yield above 25% could be detected at any concentrations, for any other amino acids but alanine. These results are in accordance with Cv-ATA having a broad substrate scope^17^ and with alanine being one of its most preferred natural substrates under physiological conditions.

### Yeast as a cell-factory for production of chiral amine

A previous study by Weber and colleagues^6^ successfully demonstrated the kinetic resolution of racemic amines by yeast expressing ATA. In that study, 25 mM racemic 1-phenylethylamine was converted to (*R*)−1-phenylethylamine with 50% conversion yield, resulting in an ee of > 99% of the *R*-enantiomer. The main reason for the efficient conversion is that the reaction is highly thermodynamically favorable towards the ketone. One way to force the reaction towards the amine is to add a high concentration of amine-donor. Our screening result reveal alanine as the most potent candidate for the conversion among amino acids: it is highly dissolved in aqueous solution, and is efficiently taken up by yeast. Based on these results, alanine was chosen as the best amine-donor candidate for this study.

Another way to make the reaction more favorable towards the amine is to enable continuous co-product removal. When yeast grows in the presence of glucose, it will rapidly convert the sugar to ethanol via pyruvate. We hypothesized that growing yeast cells would be a good production platform for chiral amines, owing to: (I) the continuous expression of ATA under physiological condition, (II) a sufficient supply of co-factor pyridoxal 5’phosphate (PLP)^8^, and (III) a continuous removal of the co-product pyruvate. To investigate for any correlation between production of MPPA and growth-state of the cell, we developed a growth-reporter gene that was introduced in yeast. If there is a correlation between the growth state of the cells (as indicated by GFP fluorescence) and the production of MPPA, it would suggest that factors influencing cell growth may also affect amine production. This information can help to optimize growth conditions and genetic modifications to improve amine production efficiency in yeast-based systems. The use of a green fluorescence protein expressed by the RPL22A promoter to follow growth has previously been demonstrated^18^. In this study we also choose RPL22A as the growth-promoter, but after screening the literature and a database for fluorescence proteins (fpbase.org) we decided to use a monomeric version of GFP (mEGFP) that contain the monomerizing A206 K mutation, to avoid any potential aggregation caused by overexpression. The growth reporter gene was assembled and introduced with crispR-cas9 in the production strain TMB4375, and the reference strains TMB4131. Both strains originate from the parental strain cen.pk2-1 C. We also included the common laboratory strain cen.pk113-7D as a control, representing the wild type phenotype. All other strains in this study were engineered from these three starting strains that contain a growth-reporter gene (Table 1, S1) as presented in the next coming sections below.

### ALT1-deletion to reduce intrinsic competition for amine-donor

**Fig. 2:**
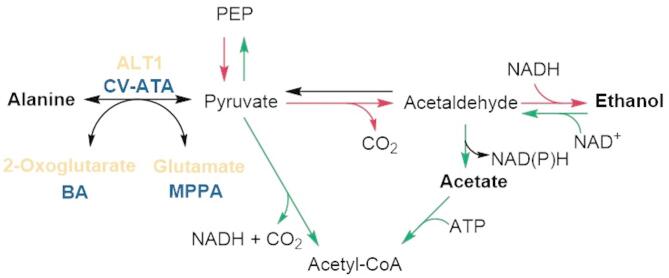
Alanine-pyruvate metabolic node in yeast.

Yeast possesses its own native alanine transaminase ALT1 that catalyzes the reversible conversion of alanine to pyruvate. ALT1 has previously been shown to be responsible for alanine catabolism and biosynthesis and is highly expressed in yeast under respiratory conditions^16,19^. α-ketoglutarate (α-KG) is the amine-acceptor, and glutamate is the corresponding co-product in this reaction (Fig. 2). Since ALT1 in the alanine-pyruvate metabolic node could potentially compete with ATA for the amine-donor, and since it is reported as a non-essential gene in yeast we decided to knock it out to improve MPPA production.

**Fig. 3:**
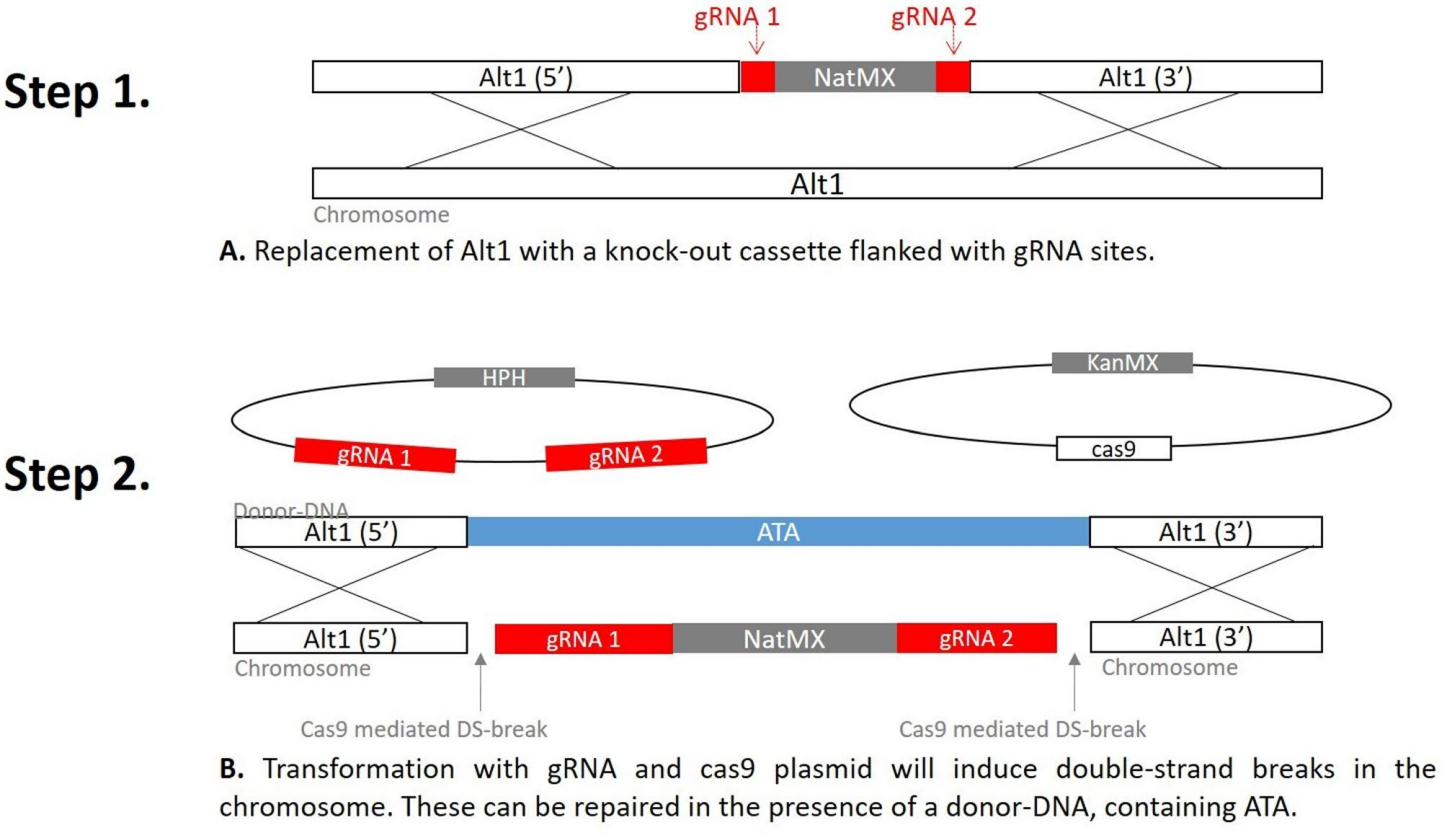
Overview of the universal two-step crispr-cas9 approach. This figure illustrates targeting the ALT1 gene in yeast. In the first step ALT1 is replaced by a nourseothricin resistance encoding gene NatMX, through homologous recombination. In the second step, NatMX that is flanked with gRNA sites is targeted by CrispR-cas9, and subsequently replaced by the ATA donor DNA in the presence of a gRNA- and Cas9-plasmid.

We also wanted to investigate the possibility to improve the production strain further by increasing the ATA gene copy number. To accomplish this, we designed a step-wise CrispR-cas9 approach to replace ALT1 with additional copies of ATA. The first step involves replacement of the ALT1 ORF with a knock-out cassette, flanked by two gRNA recognition sites, by homologous recombination. The knock-out cassette contain the gene for nourceothricin resistance (NatMX) that enable positive selection for ALT1 knock-out strains. In the second step, a duplex gRNA plasmid (TMBAHp100) was transformed together with donor-DNA, and a Cas9 plasmid^20^ pCFB2312 to introduce double-strand breaks in the genomic DNA. The donor-DNA, which contain the ATA gene can bridge the double-strand breaks and act as a template for the repair and replacement of the knock-out cassette (Fig. 3). All characterized strains with ALT1 deleted, and ALT1 replaced with a copy of ATA can be found in Table 1.

### Physiological characterization of engineered strains for MPPA production

**Fig. 4:**
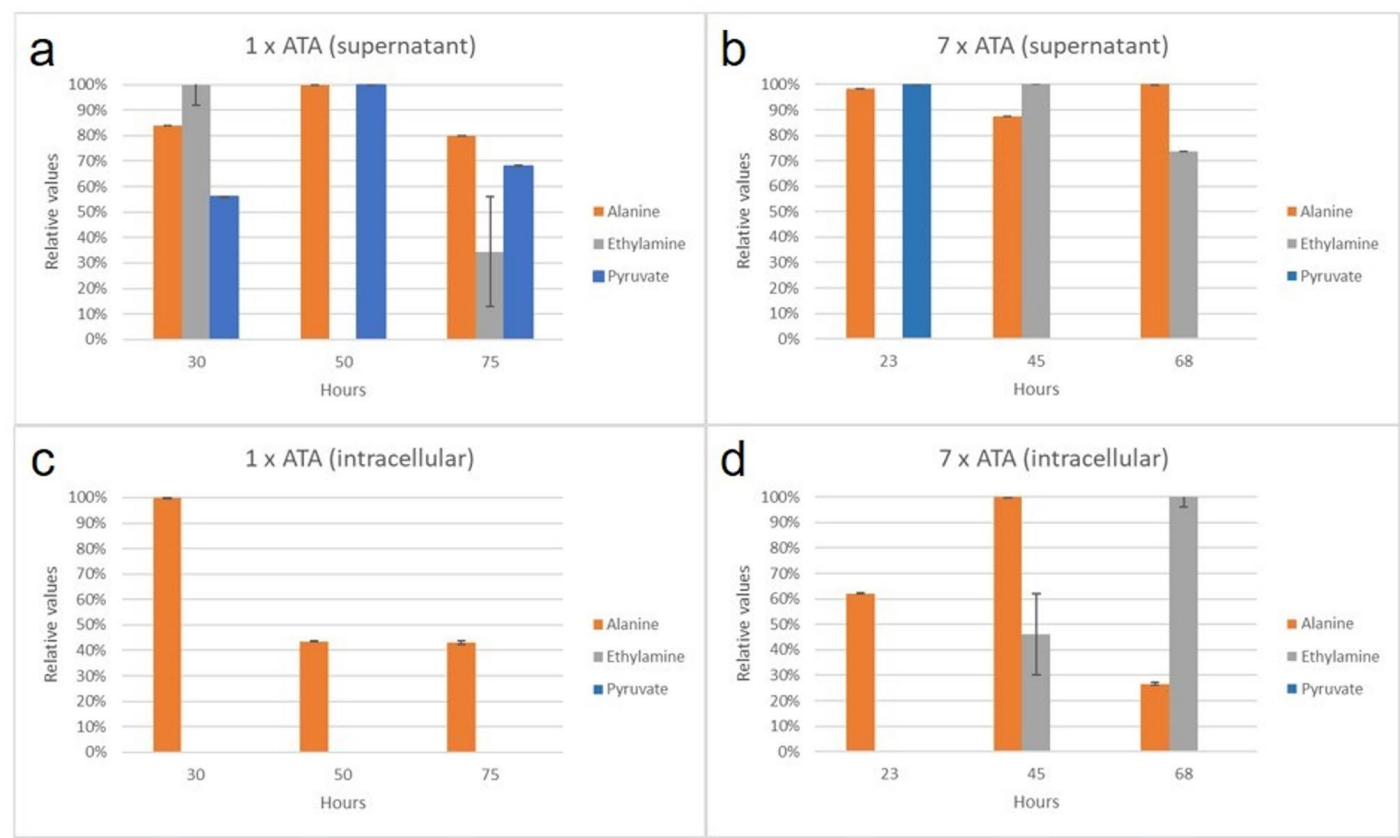
MPPA production in bioreactors. Production strains with six (6x) and seven (7x) copies of ATA, and their references with zero (0x) or one (1x) copy of ATA were tested for MPPA production. Error bars corresponds to one standard deviation of two biological replicates.

Engineered production strains and their references, with and without ALT1 deleted, and with extra copy numbers of ATA were characterized under fully controlled conditions in bioreactors for any changes in MPPA production (Fig. 4, Supplementary Table S3). Aerobic conditions were maintained throughout the experiments and cells were cultivated in synthetic minimal media^21^ supplemented with 2% glucose, 250 mM alanine, and 5 mM BA. All experiments were done under uniform condition unless stated otherwise.

As expected, MPPA could not be detected in any of the reference strains that lack ATA (TMBAH19 and TMBAH58). The production strain (TMBAH62) with seven copies (7x) of ATA gave highest MPPA yield, followed by the production strain (TMBAH25) with six copies (6x) of ATA. Deleting the native ALT1 gene and replacing it with an additional copy of ATA resulted in approximately 2.6-fold increase in MPPA yield, from 0.22 to 0.58 mol/mol (p-value = 6.3E^−2^). We also included a production strain (TMBAH59) with 6x ATA that had ALT1 deleted. This strain appears to be intermediate between the 7x and 6x strains, with a 1.8-fold increase in MPPA yield, from 0.22 to 0.40 mol/mol, but additional experiments would be required to determine the significance level. Our result suggests that both events, deletion of the native ALT1 and an additional copy of ATA, contributed to the 2.6-fold increase in MPPA yield, and that further increase in ATA copy number could result in even greater MPPA yields. We could also detect MPPA production in the cen.pk113-7D background strain (TMBAH60), which had ALT1 replaced by one copy of ATA. Even if this strain has only one copy of ATA, it still performs similar to a strain with 6 copies of ATA in the cen.pk2-1 C background. These results suggest that the cen.pk113-7D background strain provide a better platform for the production of chiral amines with transaminases. We can only speculate on the underlying mechanism, but it could for instance be related to lower inhibiting intracellular concentration of pyruvate, better uptake of extracellular alanine or higher intracellular concentration of the amino acid pool, or higher levels of co-factor PLP in the cen.pk113-7D background.

We also evaluated the intrinsic capacity of yeast to provide its own intracellular pool of amine donor for the production of MPPA. The production strain with 6 copies of ATA (TMBAH25) and its reference with 0 copies of ATA (TMBAH19) were included in the test (Supplementary Fig. S1), but without the addition of external supply of alanine. MPPA could be detected in the production strain, but not in the reference strain. These results suggest that yeast cells provide high enough intracellular amino-acid pool for MPPA production, but apparently not enough to reach the same yield as with external supply of alanine.

### ALT1 mediated growth defect on ethanol prevents MPPA production

**Fig. 5:**
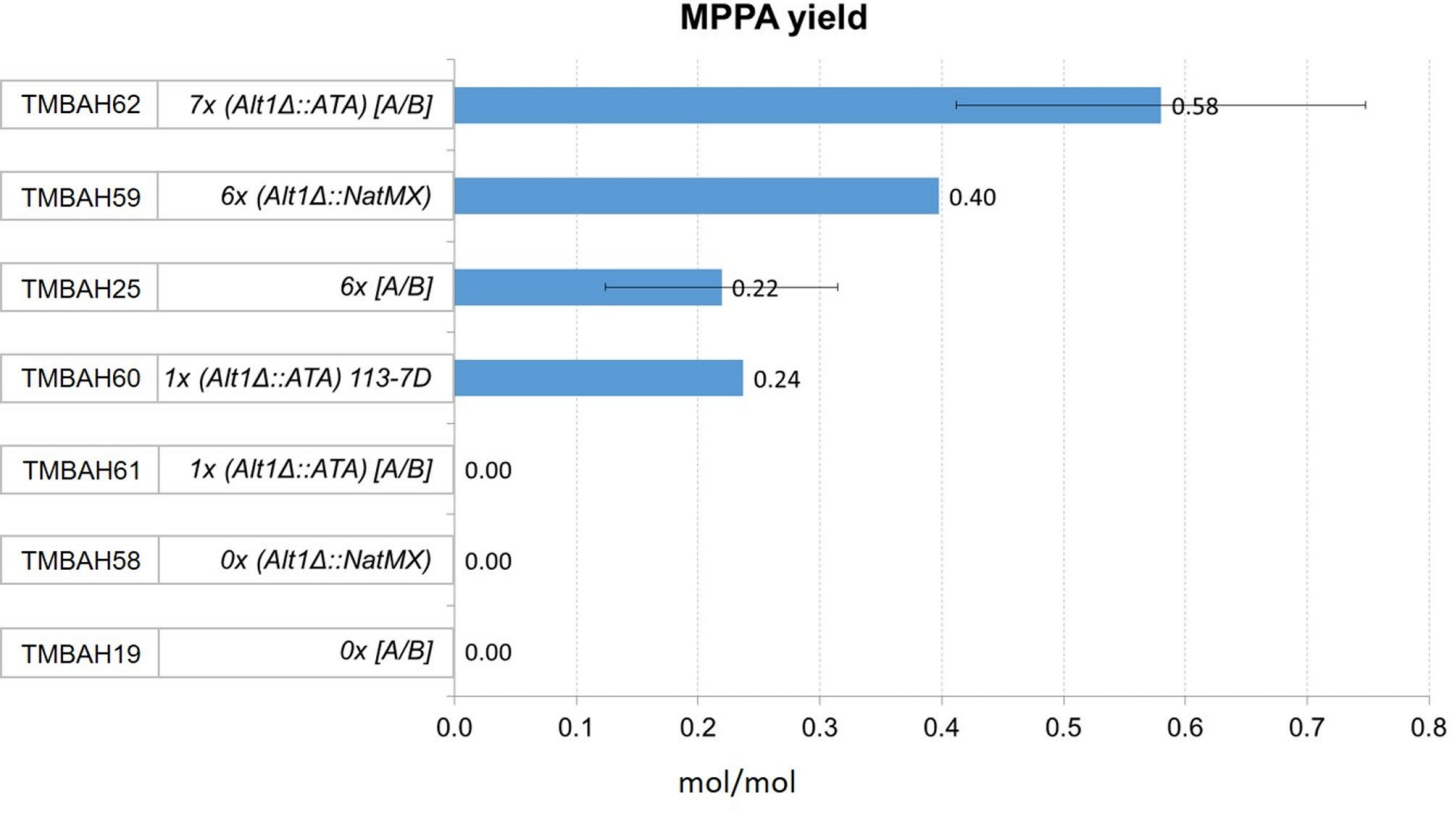
Yeast can be divided in three groups based on their growth-profile. **(A)** The first group grow on glucose and ethanol, without MPPA production. This includes the reference strain cen.pk2-1 C (TMBAH19) and the wild type strain cen.pk113-7D (TMBAH1). The second group grow on glucose, but not on ethanol, and they do not produce any MPPA. The second group includes all tested cen.pk2-1 C strains with ALT1 deleted and replaced by up to 1 copy of ATA (TMBAH58 and TMBAH61). The third group grow on glucose and ethanol, and they produce MPPA. This group includes all tested cen.pk2-1 C strains with ALT1 deleted and replaced by 6 to 7 copies of ATA (TMBAH25, TMBAH59, TMBAH62). The third group also include a cen.pk113-7D strain with ALT1 replaced by 1 copy of ATA. **(B)** Pyruvate in the culture media was quantified during growth on glucose prior to diauxic shift (green), and by the end of the experiment (orange). Extracellular pyruvate was readily detected in most cultures during growth on glucose and prior to diauxic shift, but predominantly only in ALT1 deletion strains after the diauxic shift. Biomass was determined by dry-weight during growth on glucose (grey). **(C)** Glycerol yield for different yeasts were determined under fully controlled conditions. The yields were normalized against dry weight (DW) during growth on glucose.

We could not detect any MPPA production in strain TMBAH61, with ALT1 replaced by a copy of ATA, which belong to the cen.pk2-1 C background (Fig. 4). Growth defect of ALT1 deletion strain on ethanol has been observed previously^13^. We believe this phenomenon to be related to a growth defect on ethanol as a result of deleting ALT1. Alt1 is responsible for alanine catabolism and the joint conversion of α-KG to glutamate under respiratory conditions. Decreased glutamate would affect the intracellular proteinogenic amino acid pool and growth negatively due to Alt1 deletion, and pyruvate inhibition on the conversion of α-KG to glutamate during respiratory growth. We can speculate that the overall reduced growth affects the redox balance, and the recycling of NAD(P)+, which would affect dehydrogenases and inhibit growth on ethanol at i.e. alcohol dehydrogenase 2 (ADH2) or acetaldehyde dehydrogenase (ALD2). When comparing the growth-profiles of all characterized strains they can be divided into three groups (Fig. 5a; Table 1). Group I displays the wild-type growth-profile and consist of yeast strains with a native ALT1 that do not express ATA. These yeasts grow on glucose, and after diauxic-shift, they enter a second growth-phase on ethanol. They do not produce any MPPA. Group II consists of strains that belong to the 2–1 C background with ALT1 deleted, and with ALT1 replaced with a copy of ATA. These yeasts grow on glucose, but fail to grow on ethanol as a result of ALT1 deletion. Similar to group I, they also do not produce any MPPA. Group III consists of all the remaining strains, with six and seven copies of ATA that belong to the 2–1 C background. The control strain, cen.pk113-7D with ALT1 replaced by ATA also displays the third growth profile. These yeasts grow well on glucose, and they manage to grow on ethanol and produce MPPA with the onset coinciding with the diauxic shift. From our previous work, we know that Cv-ATA is expressed at high levels in yeast during growth on both glucose and ethanol^6^. The onset of MPPA production is therefore not related to differences in expression level.

Apparently, MPPA production is inhibited during growth on the fermentable carbon-source glucose, but inhibition is released after the diauxic shift, during growth on the non-fermentable carbon-source ethanol. This phenomenon can be observed in group III yeast with up to 7 copies of ATA. Furthermore, failure to initiate growth after diauxic shift prevents yeast from producing MPPA, as observed in group II yeast. One plausible explanation of this phenomenon could be the well-known glucose repression, but despite the similarities, this explanation is unlikely since ATA is under constitutive regulation. Furthermore, the two phenomena: (1) growth defect in group II yeast, and (2) onset of MPPA production in group III yeast are most likely related, since they both occur with the onset of diauxic-shift.

### High concentrations of pyruvate can inhibit MPPA production before and after Diauxic shift

It is known that yeast rapidly ferment sugars like glucose through overflow metabolism, what results in production of overflow metabolites like pyruvate^15^. Since MPPA is only detected after diauxic-shift in the production strains belonging to group III, we hypothesize that the intracellular pyruvate concentrations could be high enough to efficiently inhibit alanine transaminase during growth on glucose. When glucose is depleted, yeast rapidly initiate a second growth phase that uses up primary and secondary overflow metabolites like ethanol and pyruvate as carbon and energy-sources^15,22^. Yeast that fail to initiate growth after diauxic-shift could in theory continue to maintain a high intracellular pyruvate concentration, which could inhibit a transaminase like ATA and prevent formation of MPPA. Thus, we hypothesized that the observed growth defect on ethanol in ALT1 deletion strains belonging to group II is the cause of, and not the result of, elevated intracellular pyruvate that continue to inhibit formation of MPPA, even after diauxic-shift.

When we measured the extracellular pyruvate that was produced during growth on glucose, the concentration in the culture media was high enough to be detected by HPLC for the majority of cultivations (Fig. 5b). For type II strains that did not produce MPPA, the pyruvate levels were ca. 0.13–0.20 g/L (or 1.7–2.3 mM), while for Type III strains, which produced MPPA, the post-diauxic levels were below or close to detection limit. The intracellular concentration can be calculated from the extracellular concentration based on the assumption that pyruvate accumulates inside the cell due to the pH gradient across the plasma membrane^23^. The distribution can be determined using the equation pH_in_ = pK_a_ + (log_10_{[(A_in_/A_out_)(1 + 10^pHout−pKa^)] − 1}), where A_in_ and A_out_ are the concentrations of the anion inside and outside the cell, respectively. The pKa for pyruvate is 2.5, and the intracellular pH is ca. 6 for glucose-starved yeast cells and ca. 7 during glucose growth^24^, while the extracellular pH is controlled at 5 in the bioreactor. This results in an accumulation factor of pyruvate_in_/pyruvate_out_ of 10–100. Consequently, the intracellular concentration for Type II strains that do not produce MPPA is calculated to be approximately 12–20 mM, which is sufficient to significantly inhibit Cv-ATA. These concentrations are in the same order of magnitude as previously observed intracellular pyruvate concentrations during respiro-fermentative growth^25^.

Inhibition of ATAs by pyruvate has been reported for the purified enzyme in several studies and seems to be a universal trait of alanine: pyruvate transaminases of different origin. At low concentrations of pyruvate, the reaction rate increases with apparent Michaelis-Menten kinetics, and above a threshold, pyruvate results in significant inhibition. The reported inhibitory concentration differs for the different enzymes (e.g., ω-Amino Acid: Pyruvate Transaminase from *Alcaligenes denitrificans* Y2k-2^26^, ω-Transaminase from *Vibrio fluvialis* JS17^27^, and (*R*)-Selective ω-Transaminase from *Mycobacterium vanbaalenii*^28^ between 8 and 80 mM, which is in range of the calculated intracellular pyruvate concentration. Reaction kinetics for Cv-ATA analyzed over a range of pyruvate concentrations have, to the best of our knowledge, not been reported previously.

Among all investigated strains, the ALT1 deletion strains TMBAH58 and TMBAH61 that belong to group II yeasts, with zero or one copy of ATA, maintain the highest pyruvate concentrations, even after diauxic shift (p-value = 1.4E^−2^) (Table S4). No significant differences in pyruvate concentrations could be detected among the same group of strains during growth on glucose (p-value = 3.5E^−1^). Furthermore, the same strains TMBAH58 and TMBAH61, which do not grow on ethanol, also display an overall reduced growth on glucose, what has resulted in a significant lower biomass yield as compared to all other strains (p-value = 1.2E^−2^) (Fig. 5b). Similar observations can be seen when comparing green fluorescence from the growth-reporter gene between group II yeasts and all other yeasts, during growth on glucose (p-value = 4.8E^−2^) (Supplementary Fig. S2a). These results are congruent with our hypothesis on inhibition of alanine transaminase by overflow metabolism.

### Alternative routes for benzylacetone metabolism

The yeast genome encodes 300 ORFs annotated as oxidoreductases, including 18 alcohol dehydrogenases (Saccharomyces genome database) that could potentially catalyze the reduction of BA to the corresponding alcohol 4-Phenylbutan-2-ol (OH). If any of the oxidoreductases has an activity towards benzylacetone, we would be able to detect OH in the culture media. The presence of OH seen in supplementary figure S3 indicates benzylacetone reductase activity in at least one of the oxidoreductases in yeast.

Interestingly OH could not be detected for any of the ALT1 deleted strains. Furthermore, there is a continuous basal consumption of BA throughout the cultivation for all strains, which is not related to growth and MPPA production (Supplementary Fig. S4). For instance, BA is continuously consumed even after diauxic-shift by yeasts belonging to group II, which do not grow on ethanol. BA is also continuously consumed even before the diauxic-shift and the onset of MPPA production in the production strains belonging to group III. In addition, BA is continuously consumed by the wildtype and reference strains that have an intact ALT1 and do not express ATA.

Since the activity of oxidoreductases are dependent on NADH and/or NADPH, we can speculate that loss of ALT1 and its role in anabolic reactions can affect growth and the redox state in cells. Reduced NADH/NAD^+^ ratio in ALT1 deletion strains could in theory inhibit the activity of a NADH dependent benzylacetone reductase and the formation of OH. The limited growth on glucose for ALT1 deletion strains belonging to group II, as indicated by the biomass yield (p-value = 1.2E^−2^), the expression of the GFP growth reporter gene (p-value = 4.8E^−2^), and the complete lack of growth on ethanol as seen on the GFP growth reporter gene (p-value = 1.2E^−2^), could be explained by this hypothesis (Table S4). Furthermore, the redox state of yeast is known to be regulated by glycerol production by the action of glycerol-3-phosphate dehydrogenase^29^. During catabolic reactions, intermediate metabolites, NADH and NADPH are produced. The cell uses NADH to drive the electron transport chain for ATP production. The intermediate metabolites act as building blocks in anabolic reactions that are fueled by ATP and NADPH. During normal growth conditions, the redox state in a cell is balanced, but if more NADH/NADPH needs to be recycled due to poor growth, yeast could in theory compensate for that by increasing glycerol production. When comparing the glycerol yield among strains grown on glucose, a highly significant increase (p-value = 1.2E^−2^) can be detected among all slow growing yeasts belonging to group II (Fig. 5c, Supplementary Table S4).

It is important to note that yeast has been reported to produce glycerol under two additional conditions related to stress. These are (I) at pH 7 and above, and (II) under high osmotic stress^29,30^. It is however unlikely that the remarkable differences in glycerol yield would be caused by these two stresses, since the cultivations were conducted under fully controlled, uniform and optimum growth conditions in bioreactors.

While the activity of ATA and MPPA formation is exclusively restricted to ethanol growth-phase in group III yeasts, the concentration of BA is continuously reduced in the culture media throughout the experiment (Supplementary Fig. S4). Since BA is an oily substance with a tendency to stick to various surfaces, and its continuous reduction in the culture media could be correlated to biomass formation, we decided to investigate this further. Indeed, when we tested a high biomass concentration (OD 25), we could detect the effect of BA bound to biomass (Supplementary Fig. S5). This effect could be as high as 10% of the observed loss of BA in all fermentations. However, since the unexplained loss of BA could be as high as 80% among MPPA producing strains (Supplementary Fig. S6), the absorption of BA to biomass can only partly explain the continuous reduction of BA throughout all fermentations.

Our results suggest that yeast converts BA to additional compounds other than MPPA and OH, and there must be other competing reactions for BA, and possibly alanine. To investigate this further, we decided to identify any unknown product by NMR using ^15^N labelled alanine as amine donor, and ^13^C labelled glucose as the only carbon source.

We first scaled down the 250 ml bioreactor experiments to 25 ml in baffled Erlenmeyer flasks, and finally to 1,5 ml in mini-vials. Two strains representative of group II, and group III yeasts were included in the experiment. These two strains were the reference strain (TMBAH61) with one copy of ATA, and the production strain (TMBAH62) with 7 copies of ATA. No changes in growth-profile could be detected for any strain during the down-scaling process (Supplementary Fig. S7, Fig. S8). In other words, the reference strain displayed the growth-profile typical of group II yeasts, and the production strain followed the growth-profile of group III yeasts, at all three culture volumes tested. Three time-points were taken for NMR measurements, as can be seen in figures S7 and S8. The first time-point was taken during glucose growth for both experiments after 23 h (TMBAH61) and 30 h (TMBAH62). The second time-point was taken after 45 h (TMBAH61) when glucose was depleted, and after 50 h (TMBAH62) during ethanol growth. The third time-point was taken after 68 h (TMBAH61), and after 75 h (TMBAH62) after ethanol was depleted.

**Fig. 6:**
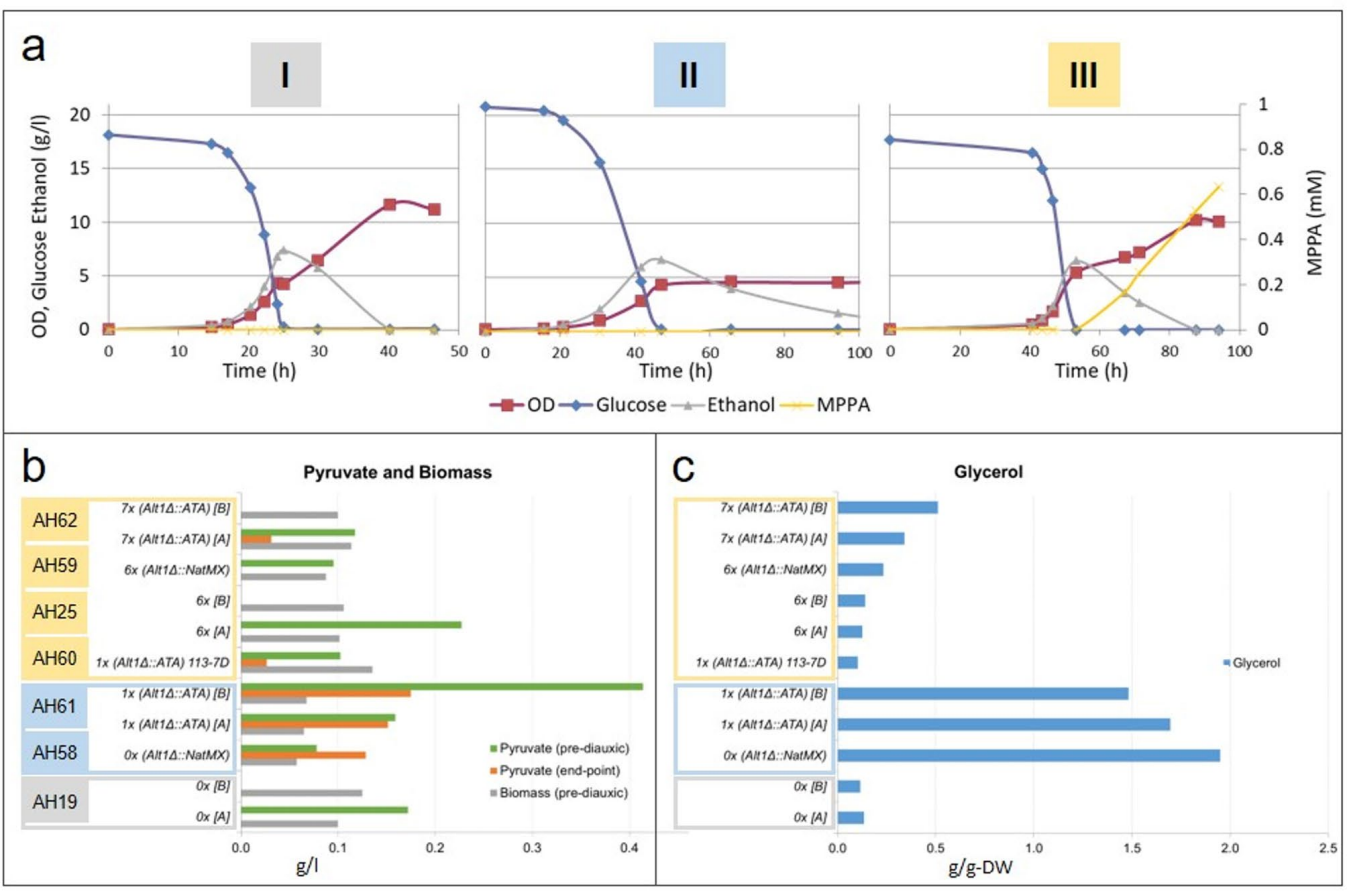
Detection of ^15^N labelled alanine and ethylamine, and ^13^C labelled pyruvate in the reference strain with one copy of ATA (TMBAH61), and the production strain with 7 copies of ATA (TMBAH62). Compounds were detected in extracellular supernatant (A and B), as well as intracellular samples (C and D).

The ^15^N spectra revealed three major peaks of which two were known, alanine and MPPA (Supplementary Fig. S9). The third unknown peak was successfully identified as ethylamine (C_2_H_7_N). Alanine and ethylamine could also be detected in the ^13^C spectra, along with pyruvate. Alanine was detected in all three time points, both intracellularly and in the culture media for both strains (Fig. 6). Our results also demonstrate that alanine was not depleted during the experiments, since it was detected in high abundance, in all three time points. MPPA is only visible in ^15^N spectra, which confirm that BA added to the culture media is the only source for MPPA production.

Unfortunately, pyruvate could not be detected in any of the intracellular samples, which means that more cells were needed for the extraction, or that a more efficient extraction method for this central carbon- metabolite is required (Fig. 6). The pyruvate concentration in the culture media was however high enough to be detected by NMR. Pyruvate could be detected at the first time-point, during glucose growth phase for both strains, but only in the last two time-points for the reference strain. This result is congruent with our HPLC data (Fig. 5b) and further confirms our hypothesis on inhibition of alanine transaminase by overflow metabolism. Ethylamine was detected intracellularly only in the production strain (TMBAH62) at the two last time-points, which suggested that ethylamine was produced from acetaldehyde by ATA. To shed more light on whether Cv-ATA could indeed have produced ethylamine, we analyzed the activity of the purified enzyme using an alanine transaminase activity assay with ethylamine or MPPA as amine donor. While MPPA was readily accepted as substrate by the purified enzyme, we could not detect any activity with ethylamine as amine donor (Supplementary Figure S10). Furthermore, no significant transaminase activity towards ethylamine or MPPA was detected in cell-free extract from the cen.pk background strain. These findings suggest that ethylamine production is likely due to native yeast enzyme activity. Future studies could focus on identifying and characterizing these native enzymes to better understand their contributions to the overall metabolic network.

## Conclusions

In conclusion, our study demonstrates that metabolically engineered baker’s yeast is an effective catalyst for producing chiral amines from prochiral ketones. We found that using ethanol as a co-substrate, rather than glucose, significantly enhances amine production, likely due to lower intracellular pyruvate levels. Furthermore, replacing the native alanine aminotransferase (ALT1) with omega transaminase from *Chromobacterium violaceum* (cv-ATA) markedly improved production by reducing competition for alanine utilization. Under optimal conditions, the bioconversion of benzylacetone to (*S*)−1-methyl-3-phenylpropylamine achieved high yield. These findings underscore the potential of engineered yeast and optimized metabolic pathways to advance the efficient production of valuable chiral amines.

## Methods

### General molecular work

All PCR reactions were done using phusion (Thermo Scientific) unless stated otherwise, and synthetized DNA oligos were ordered from Eurofins Genomics. All PCR purifications were done with GeneJET PCR purification kit (Thermo Scientific) and all gel-extractions were done with low melt agarose (Bio-Rad) and GeneJET gel extraction kit (Thermo Scientific). All plasmid purifications were done using GeneJET plasmid miniprep kit (Thermo Scientific). All ligations were done with NEBuilder (New England Biolabs), and all yeast transformations were done using Li-Acetate heat-shot protocol^31^.

### Strain development – reporter strains

Reporter strains were constructed to monitor growth during process optimization for conversion of ketone BA to chiral amine MPPA. A recombinant growth reporter gene consisting of mEGFP and the CYC1 terminator under the control of a RPL22 A promoter was assembled and sub-cloned in the pCfB3034 plasmid. The reporter gene was subsequently introduced in the X-3 locus of yeast strains TMB4375 that already contain 6 copies of ATA^6^, the reference TMB4131, and a wild type strain cen.pk.113-7D (Table 1, S1) using the CrispR-cas9 easy clone approach^20^. We upgraded a previously developed growth reporter system^18^ by using a monoclonal yeast enhanced version of GFP (mEGFP) that contains the monomerizing A206 K mutation for improved signal and reduced chance of protein aggregation, due to overexpression^32,33^. The RPL22a promoter fragment was amplified with primer pairs 5’X-3_RPL22a Fw and 5’mEGFP_RPL22a Rv using genomic cen.pk113-7D as template DNA. The mEGFP ORF was amplified with primer pairs mEGFP Fw and mEGFP Rv using plasmid mEGFP-FKBP(M)x4 as template DNA. The plasmid was a gift from Benjamin Glick (Addgene plasmid #85004; http://n2t.net/addgene:85004; RRID: Addgene_85004). A CYC1 terminator was also amplified from that same plasmid with primer pairs 5’mEGFP_CYC1 Fw and 3’X-3_CYC1 Rv. Prior to assembling the recombinant growth reporter gene and cloning it, a plasmid backbone was prepared by digesting pCfB3034 with restriction enzymes SbfI-HF and BstAPI (New England Biolabs) following the manufacturers recommendations. The digested plasmid was gel-purified from low melt agarose. An initial attempt to assemble all fragments in an equimolar fraction using NEBuilder with 100ng of SbfI and BstAPI double-digested plasmid backbone resulted in no positives. This was resolved by first assembling all growth reporter gene fragments (RPL22A, mEGFP, and CYC1) in an equimolar ratio using NEBuilder. The assembled construct was then amplified with PCR using the flanking primer pairs 5’X-3_RPL22a Fw and 3’X-3_CYC1 Rv. The NEBuilder reaction mixture was diluted 100 times and used as a source for template DNA. The fragment of correct size was gel-purified from low melt agarose, and cloned in a final step with SbfI and BstAPI double-digested pCfB3034 plasmid backbone using NEBuilder, following the manufacturers recommendations. Positive transformants were screened for in a first step by double digestion using SwaI and FspI (New England Biolabs) and finally sequence verified (Eurofins Genomics).

Donor DNA for CrispR-cas9 transformation was prepared from one positive transformant, cultured overnight in a shaking incubator at 37 ^o^C in 10 ml LB supplemented with ampicillin. Plasmid was extracted and donor DNA was excised with SmiI (New England Biolabs). After PCR purification, 1 ug of donor DNA was added together with 250 ng of gRNA (pCfB3041) and used to transform yeast that already contain the Cas9 plasmid pCFB2312^20^. Transformants expressing both gRNA and Cas9 were selected for on YPD 2% agar supplemented with nourseothricin (100 ug/ml) and geneticin (200 ug/ml). Positive transformants were verified by fluorescence and full length insert by PCR using primer pairs X-3 ver-Fw and X-3 ver-Rv. Three positives of each strain were kept as −80 ^o^C glycerol stock.

### Amine (MPPA) production strains (ATA 1x, 6x, 7x)

CrispR-cas9 constructs were designed for replacement of yeast native ALT1 allele with an additional copy of ATA in three growth reporter strains. These were (I) the cen.pk113-7D reference strain TMBAH13, (II) the cen.pk2-1 C reference strain TMBAH19, and (III) the cen.pk2-1 C production strain TMBAH25 that already contain 6 copies of ATA (Table 1, S1). In the first step, the native ALT1 allele was replaced by a nourseothricin resistance encoding gene (NatMX) through homologous recombination. In the second step, the NatMX-knockout cassette, which is flanked with gRNA sites was targeted by Crispr-cas9, and subsequently replaced with the ATA gene (illustrated in Fig. 3).

The NatMX-knockout cassette was constructed by PCR, using primer pairs 5’ALT1_NAT Fw and 3’ALT1_NAT Rv (Table S5), and plasmid pCfB3041 as template DNA. Correct PCR product was verified for its integrity with 1% agarose gel-electrophoresis, and subsequently PCR purified. Yeast were transformed with 0.5 ug of purified PCR product, and knock-out transformants were selected for on YPD 2% agar plates supplemented with nourseothricin (100 ug/ml).

A duplex gRNA plasmid that can be selected for on hygromycin, and that target both flanks of the NatMX-knockout cassette was constructed. First a plasmid back-bone without the existing nourseothricin resistance encoding gene was prepared from digesting pCfB3043 with SphI-HF and NcoI-HF (New England Biolabs) followed by gel-purification. A hygromycin resistance encoding ORF (HPH) was amplified with primer pairs HPH-Fw and HPH-Rv using pRS305H^34^ as template DNA, and subsequently PCR purified. Both fragments were ligated in a 2:1 ratio with 50 ng of digested plasmid backbone using NEBuilder. Positive clones were verified by PCR, using primer pairs pCfB3043-Fw and pCfB3043-Rv, followed by PCR purification and integrity check on low melt agarose gel. A universal gRNA cloning site, used for inserting gRNA either as singlets or for multiplexing, was designed and subsequently ordered from Eurofins Genomics and delivered as a plasmid (Table S5). The sub-cloned fragment was amplified with PCR using primer pairs cloningsite-Fw and cloningsite-Rv. The reaction was PCR purified, verified by gel-electrophoresis and subsequently ligated in a 2:1 ratio with 50 ng of PCR amplified plasmid backbone using NEBuilder. Positive transformants were sequence verified (Eurofins Genomics) with primer pairs gRNA_seq-Fw and gRNA_seq-Rv. One positive plasmid was digested with XmajI and XhoI (Thermo Scientific) followed by gel-extraction. A duplex gRNA fragment was designed and subsequently ordered from Eurofins Genomics, and delivered as a plasmid (Table S5). The sub-cloned fragment was amplified with PCR, using primer pairs gRNA-Fw and gRNA-Rv. The reaction was PCR purified and ligated in a 2:1 ratio with 50 ng of the double-digested plasmid backbone. Positive transformants were sequence verified (Eurofins Genomics) with primer pairs gRNA_seq-Fw and gRNA_seq-Rv and two positive clones were stored in glycerol stock at −80 ^o^C.

The donor DNA, which consist of the ATA gene flanked with 50 bp long sequences that are homologous to the upstream and downstream sequences of genomic ALT1, was constructed by PCR using pNW12^6^ as template. The primer pairs were 5’ALT1_cvATA-Fw and 3’ALT1_cvATA-Rv. PCR product was verified with 1% agarose gel electrophoresis, followed by PCR purification and later used as donor DNA.

Yeast strains that had their native alanine transaminase (ALT1) replaced by a NatMX-knockout cassette were transformed with 700 ng of Cas9 plasmid pCFB2312, 500 ng duplex gRNA and 500 ng of donor DNA. Transformants containing both plasmids were selected for on YPD 2% agar plates supplemented with geneticin (200 ug/ml) and hygromycin (300 ug/ml). Positive transformants that had lost the NatMX-gene and gained one extra copy of ATA, were verified by colony PCR using Q5 polymerase and GC-enhancer (New England Biolabs) and primer pairs ALT1-Fw and ALT1-Rv.

### Amino acid screening

Different amino acids were tested for how well they function as amine-donors for ATA expressed in yeast. Amino acids were screened at three concentrations, 250 mM, 500 mM, and 1 M. A total of ten amino acids can be dissolved at 250 mM and they were all included in this study (Fig. 1b). Out of these ten amino acids, eight could be dissolved at 500 mM, and five out of these could be dissolved at the highest concentration 1 M. The only buffer was YP, and the start and end pH was determined at the highest amino acid concentration. Single colonies of yeast from agar plates were used to inoculate 3 ml of YP 2% glucose broth in 15 ml Falcon tubes. The cultures were incubated overnight at 30 ^o^C, and at 250 rpm. The overnight cultures were diluted approximately 500 times in 50 ml of YP 5% glucose broth, in baffled 250 ml Erlenmeyer flasks, equipped with cotton plugs to ensure aerobic condition. The cultures were then incubated at 30 ^o^C in a shake-incubator at 250 rpm overnight, for approximately 16 h, until reaching the post-exponential phase of OD_600_ between 16 and 18. Optical density was measured and the volume needed, to dissolve in 10 ml of OD 25, were transferred to a 50 ml falcon tube and stored on ice. Cells were concentrated by centrifugation at 3000 g, and the pellets were resuspended to a final volume of 10 ml ice-cold YP 5% glucose broth, supplemented with 5 mM benzylacetone (BA) and the amino acid. Cell-suspensions were transferred to 50 ml serum flasks equipped with a 20 mm magnetic stirrer, and rubber-lids. The lids were perforated with a syringe needle, closed with a cotton plug, to ensure aerobic conditions. The serum flasks were incubated at 30 ^o^C in a water bath equipped with magnetic stirrers set to 250 rpm. 800 ul samples for HPLC were taken at the start, and at the end of each experiment after 6 days. Samples were transferred to 1,5 ml microcentrifuge tubes that were kept on ice until centrifugation at 9000 g for 3 min. Supernatant were filtered through 0,2 micron PTFE filters, and stored at −20 ^o^C until further analysis with HPLC.

### Characterization in bioreactors

Yeasts were characterized for MPPA production in minifors bioreactors (INFORS HT) with a working volume of 250 ml. All cultivations were done at 30 ^o^C and in duplicates, unless stated otherwise. The stirrer speed was set automatically varying from 200 to 1000 rpm to maintain aerobic condition, by keeping the dissolved oxygen concentration above 30% throughout the whole experiment. Dissolved oxygen was monitored with an InPro 6800 S sensor (Mettler Toledo). pH was maintained at 5 (± 0.5 units) by KOH (1 M) and H_2_SO_4_ (0.5 M), monitored with a 405-DPAS-SC-K8S/225 (Mettler Toledo) sensor. CO_2_ and O_2_ levels were determined in the gas outflow with gas analyzers BC-CO2 and BCP-O2 (BlueSens). All cultivations were done in synthetic minimal media^21^ using 2% glucose as the sole carbon source. Overnight cultures were done in YP 2% glucose broth, and all pre-inoculums were washed in synthetic media once and diluted approximately 250 times when inoculating a bioreactor. This resulted in approximately 250 times higher biomass by the end of each fermentation. Samples were taken at different time points for HPLC measurements, and growth was followed by OD_600_ and dry-weight measurement as previously described^22^. Growth was also monitored on single-cell level with flow cytometry by detecting green fluorescence in live cells, which was expressed by a growth-reporter gene.

### BA absorption to biomass

Overnight culture of the wild type cen.pk113-7D strain was washed in ice-cold water, and concentrated to OD_600_ 25 in 2 ml minimal media^21^ supplemented with 5 mM BA. The sample was thoroughly mixed using a pipette, and 1 ml was serial diluted three times to a final OD_600_ of 3 in 2 ml microcentrifuge tubes. The serial diluted samples were then centrifuged 3 min at maximum speed and stored on ice. Supernatant were filtered through 0,2 micron PTFE filters and kept on ice until HPLC measurements.

### Cultures for NMR

Experiments were downscaled from 250 ml in bioreactors, to 25 ml in a 100 ml baffled Erlenmeyer flasks, and finally to 1,5 ml in a 5 ml mini-vials equipped with a 10 mm magnetic stirrer. All cultures were grown in same synthetic media with same concentrations of supplements as in the bioreactor experiments, apart from alanine and glucose for NMR measurements ^15^N alanine, and ^13^C glucose were used for cultivations in mini-vials. The starting cultures for Erlenmeyer flasks and mini-vial experiments were prepared similarly to those for the bioreactor. Yeast pre-cultures were grown overnight in YP 2% glucose, washed in synthetic media, and diluted 250 times in either Erlenmeyer flasks or to OD 0,2 in mini-vials. Erlenmeyer flask were closed with cotton-stoppers, and the cultures were incubated at 30 ^o^C in a shake incubator, set to 250 rpm to ensure aerobic conditions. Mini-vials were closed with cotton stoppers and cultures were incubated in a 30 ^o^C water bath, equipped with magnetic stirrers that were set to 250 rpm to ensure aerobic conditions. Sampling for OD, DW and HPLC measurements from Erlenmeyer flask cultures were done in the same way as for the bioreactors. Samples from mini-vials were taken at three time-points, for each strain and experiment. Three replicates were run in parallel for each strain. At each time-point, 3 × 300 ul were taken from one replicate and separated into three 1,5 ml microcentrifuge tubes kept on ice, and immediately centrifuged at 10 000 g for 1 min at 4 ^o^C. Supernatant were pooled and filtered through a 0,2 micron PTFE filter. 500 ul of the filtered supernatant was added to a 1,5 ml microcentrifuge tube, and the remainder to another microcentrifuge tube that was kept at −20 ^o^C for HPLC measurements. The 500 ul supernatant sample was snap frozen in dry-ice mixed with ethanol, and stored long-term at −80 ^o^C until NMR measurements. Cell- pellets were first washed in 500 ul ice-cold minimal media, and then snap frozen in dry-ice and ethanol, and stored long-term at −80 ^o^C.

Cell extracts were prepared for NMR by dissolving frozen pellets in 600 ul of ice-cold 80% methanol^35^. Samples were kept on ice for 30 min, then filtered through a 0,2 micron PTFE filter, and purged with N_2_ for 30 min on ice, and finally lyophilized for 4 h. Frozen 500 ul filtered supernatant samples were directly lyophilized for 4 h. All samples were kept at −80 ^o^C prior to NMR measurements.

### Flow cytometry

Green fluorescence was measured with a BD Accuri C6 flow cytometer equipped with a 510/15 nm detection filter (BD Biosciences). Culture samples were diluted in phosphate buffered saline solution to an OD_600_ of approximately 0,5. For each sample, 5 ul propidium iodide (250 mg/l) was added to stain dead cells. Samples were incubated in room temperature, protected from light for 10 min before measurement with the flow cytometer. Cells were detected at a flowrate of 35 µl/min, and events above a threshold of 80 000 were measured with forward scatter height (FSC—H). A total of 10 000 events were registered for each sample for further data-analysis using a script compiled in Matlab for this study. The script is available for downloading the following GitHub repository: https://github.com/MicrobialEngineeringGroupTMB/.

This script applies combinations of four different filters on the data-set, one static, one dynamic and two that are user-specified. The first static filter retains non-zero events that are considered as cells in the green (FL1-H) and the red (FL3-H) channels. The second filter separates single-cells from the rest by applying a user-defined cut-off value on the width-channel. Under uniform conditions in a bioreactor, this filter is usually optimized once for each strain. The third filter is used to define green-fluorescent sub-populations by applying a user-defined cut-off value on the FL1-H channel. Under uniform conditions in bioreactors, this filter is usually set once for the specific biosensor used. The fourth filter automatically separates live cells from the rest of the data-set by modeling the sub-population consisting of live-cells in the FL3-H channel, with the k-density function in Matlab. The rationale behind using an automated sorting algorithm for this parameter is because it is the only one expected to vary, even within uniform culture conditions and the same strain. This parameter is for example dependent on the incubation time of PI, or in other words, when exactly the sample is run in the flow cytometer after addition of PI. The time might vary depending on the sample size at a specific time-point. By applying the defined gates in different combinations for each time-series, we could define subsets of relevant subpopulations that might not be detected otherwise by other statistical or visualization methods. For example, subpopulations of (I) Live cells (II) Live Single cells (III) Live Single GFP positive cells and (IV) Live Single GFP negative cells, and their corresponding counterparts were followed for each cultivation.

### HPLC

Glucose, pyruvate, succinate, glycerol, acetate, and ethanol were quantified by high-performance liquid chromatography binary pump 1525 (Waters) equipped with a refractive index detector 10 A (Shimadzu) and a 300*7.8 mm aminex HPX-87 H column (Bio-Rad). The mobile phase was 5 mM H_2_SO_4_ with a constant flow rate of 0.60 ml/min through a column temperature of 60 ^o^C.

4-Phenylbutan-2-one (BA), 4-Phenylbutan-2-amine (MPPA) and 4-Phenylbutan-2-ol (OH) were quantified by high-performance liquid chromatography (Dionex 3000 RS with UV 260 nm detection) and an Evo C18 2.6 µ 50 × 2.1 mm column (Phenomenex). The mobile phase was 80% water containing NaOH pH 11.3 and 20% acetonitrile, with a constant rate of 0.5 ml/min through a column temperature of 30 ^o^C.

### NMR

Lyophilized samples were dissolved in 80% v/v of 100 mM acetate buffer, pH 4 and 20% v/v acetone d_6_^35^. In order to identify compounds, four reference samples were prepared that contained MPPA, BA, pyruvate and ethylamine. All NMR experiments were performed on a 600 MHz Agilent VNMRS DirectDrive spectrometer. Heteronuclear single-quantum correlation (HSQC) experiments^36^ were performed at temperatures of 298 K and 268 K for ^13^C and ^15^N nuclei, respectively. In the ^15^N-HSQC, the spectral widths were 13 ppm covered by 1024 data points for 1 H, and 30 ppm covered by 256 data points for ^15^N. In the ^13^C-HSQC, the spectral widths were 9.5 ppm covered by 1024 points for ^1^ H, and 140 ppm covered by 256 points for ^13^C. All spectra were processed using NMRPipe^37^, employing solvent filtering, squared cosine apodization, and zero filling to twice the number of data points. All spectra were analyzed in the CCPNmr program suite^38^. Peak volumes were integrated using PINT^39,40^.

### Transaminase enzyme assay

Pure Cv-ATA (kindly provided by Dr. Isabela Mendes Bonfim) and cell-free yeast extract from Cen.PK 113-7D grown on ethanol was evaluated for transaminase activity with ethylamine, and MPPA as substrate. The reaction was performed at 30 degrees in HEPES-buffer at pH 7, containing 2 mg/L Cv-ATA, 1 mM PLP, 500 µM pyruvate, and 5 mM MPPA or ethylamine. The reaction ran for 30 min and was quenched by boiling the sample for 10 min in a heat block. The concentration of alanine was measured with an Alanine Kit (Cell Biolabs) according to the instructions from the manufacturer.

### Statistical analysis

Statistical tests were done with R (version 4.3.0). Several growth parameters and central carbon metabolites were investigated for significant differences among groups with Mann-Whitney U test. The investigated parameters and the test results are summarized in Table S4. Even if equal variance cannot be assumed for any of the investigated parameters, as a result from failed Bartlett and Levene test, similar conclusions are still supported by Welch-test. MPPA yield comparison among strains were tested for normal distribution (QQ-plot) and equal variance (F-test). Only when passing both tests, a one-sided and two-sample T-test was calculated to test for significance.

## Electronic supplementary material

Below is the link to the electronic supplementary material.

Supplementary Material 1

Supplementary Material 2

Supplementary Material 3

## Data Availability

DNA sequences in this study are available in the Supplementary Information. Other relevant datasets used and/or analyzed during the study can be made available upon reasonable request to the corresponding author.

## Code availability

The custom made Matlab script that was used to analyze all flowcytometry data in this study can be downloaded from the following GitHub repository: https://github.com/MicrobialEngineeringGroupTMB/.
