# Supplementary material for: Biocatalytic reductive amination with CRISPR-Cas9 engineered yeast
Source: Sci Rep. 2025 May 15;15:16972. doi: 10.1038/s41598-025-01182-0 (PMC12081890; doi:10.1038/s41598-025-01182-0)

Figure S4
p1

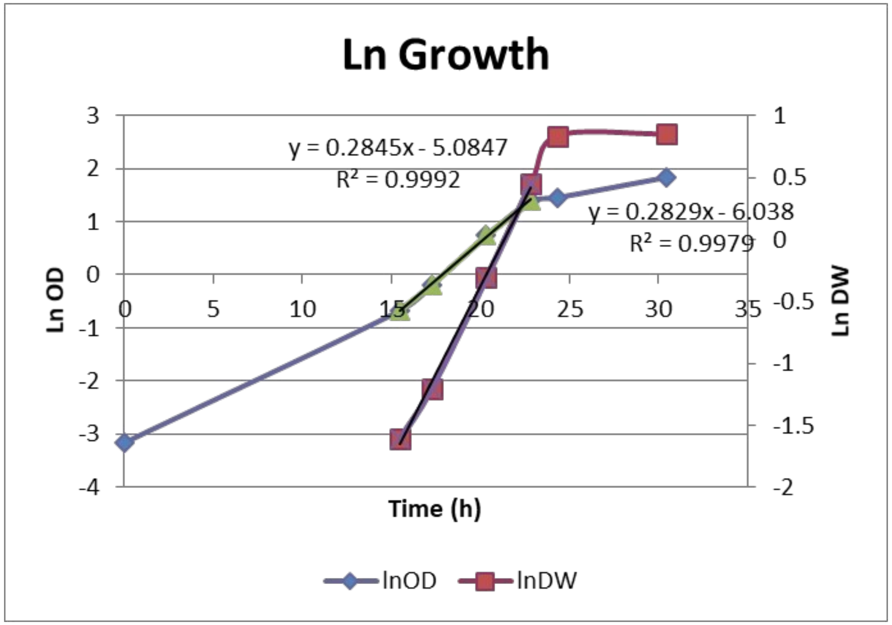

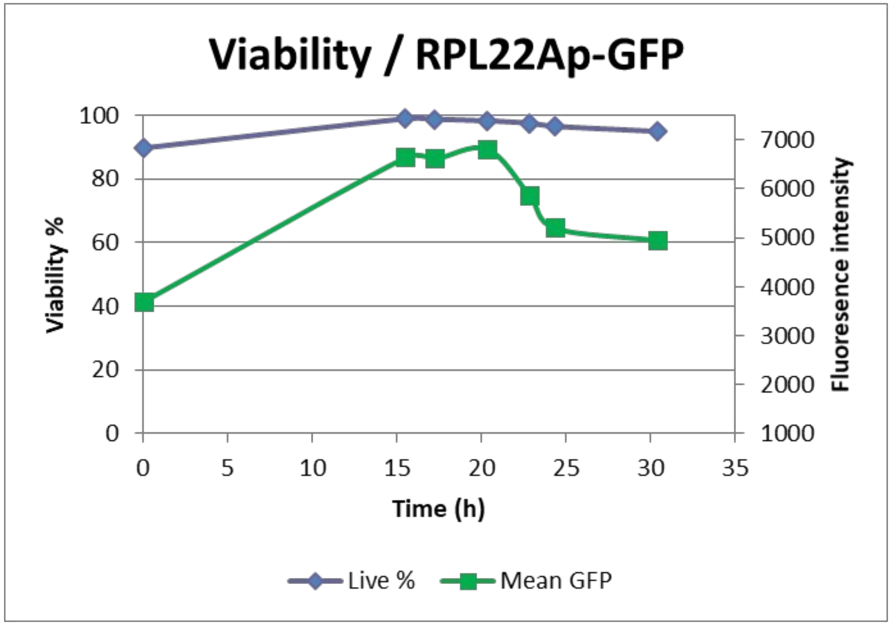

TMBAH19 [A] 0x

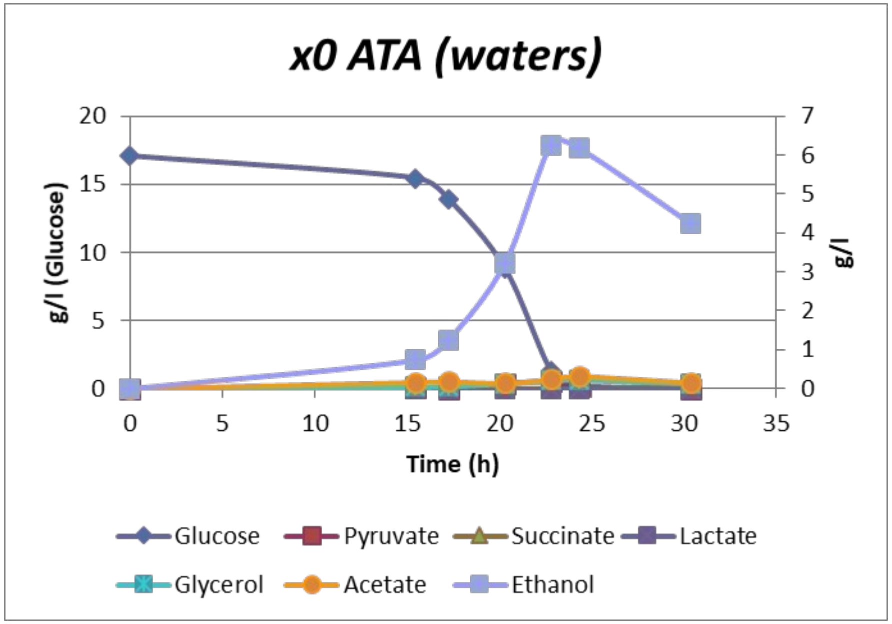

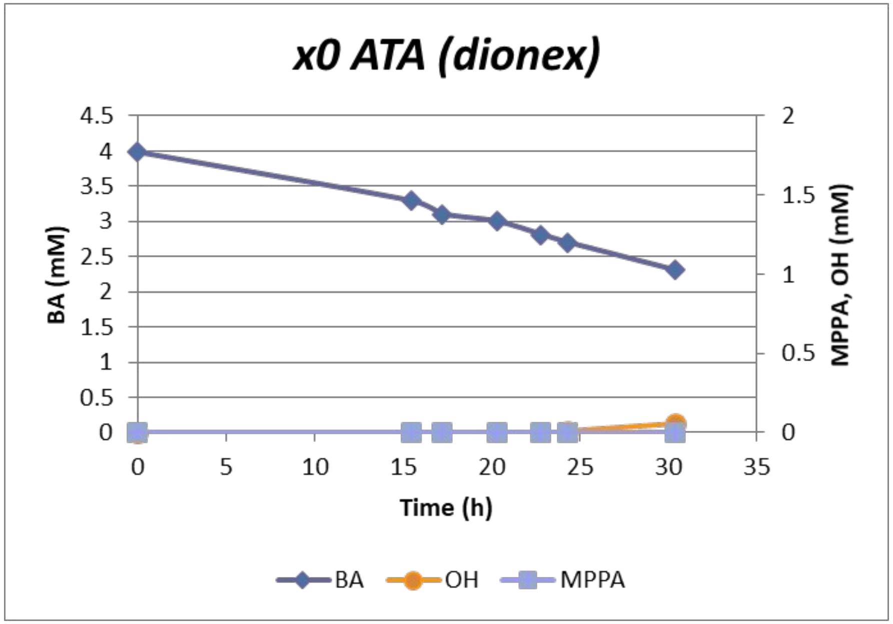

Figure S4
p2

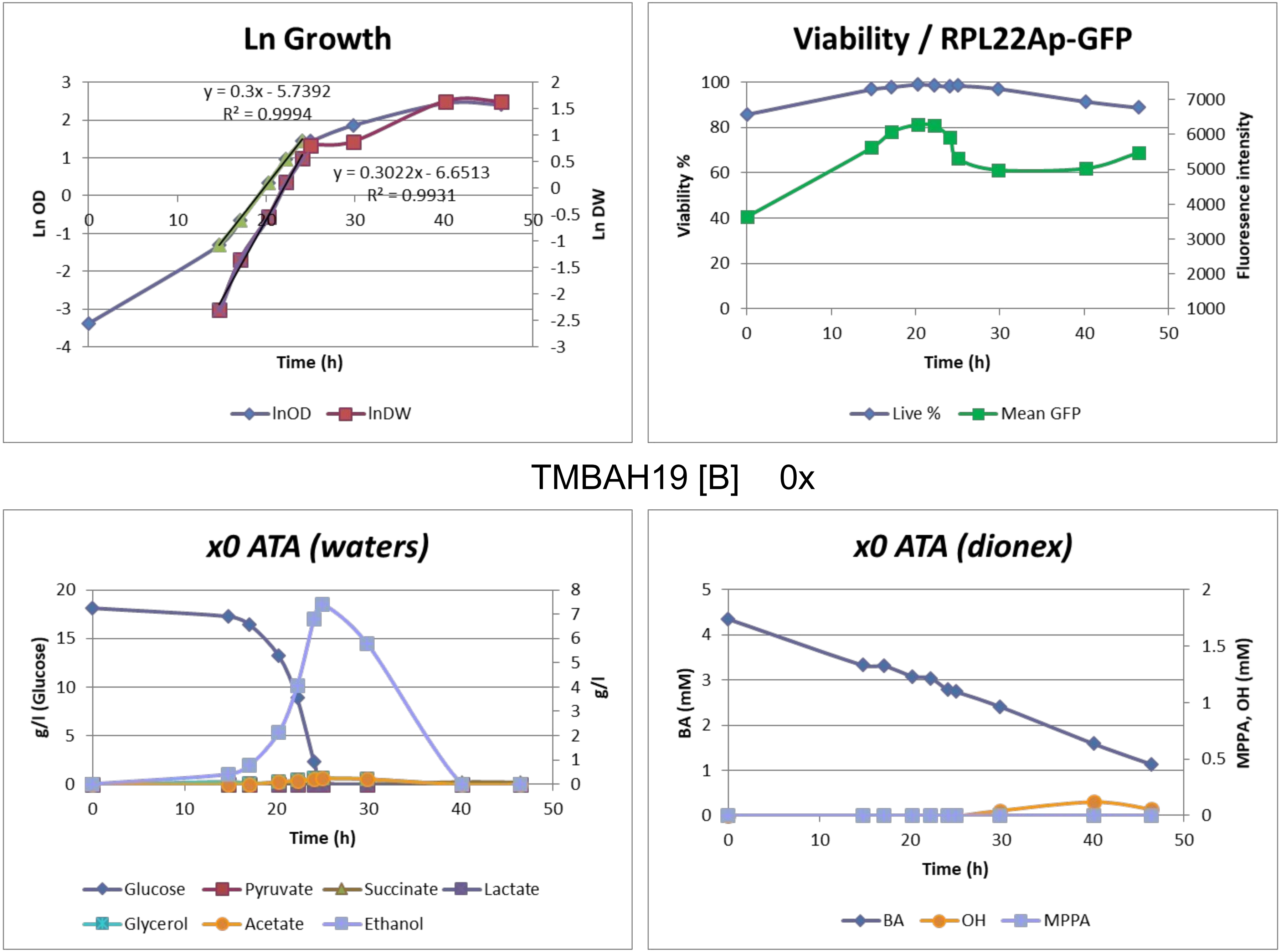
Ln Growth
y = 0.3x - 5.7392
R² = 0.9994
y = 0.3022x - 6.6513
R² = 0.9931
Ln OD
Ln DW
Time (h)
lnOD
lnDW
Viability / RPL22Ap-GFP
Viability %
Fluoresence intensity
Time (h)
Live %
Mean GFP
TMBAH19 [B] 0x
x0 ATA (waters)
g/l (Glucose)
g/l
Time (h)
Glucose
Pyruvate
Succinate
Lactate
Glycerol
Acetate
Ethanol
x0 ATA (dionex)
BA (mM)
MPPA, OH (mM)
Time (h)
BA
OH
MPPA

Figure S4
p3

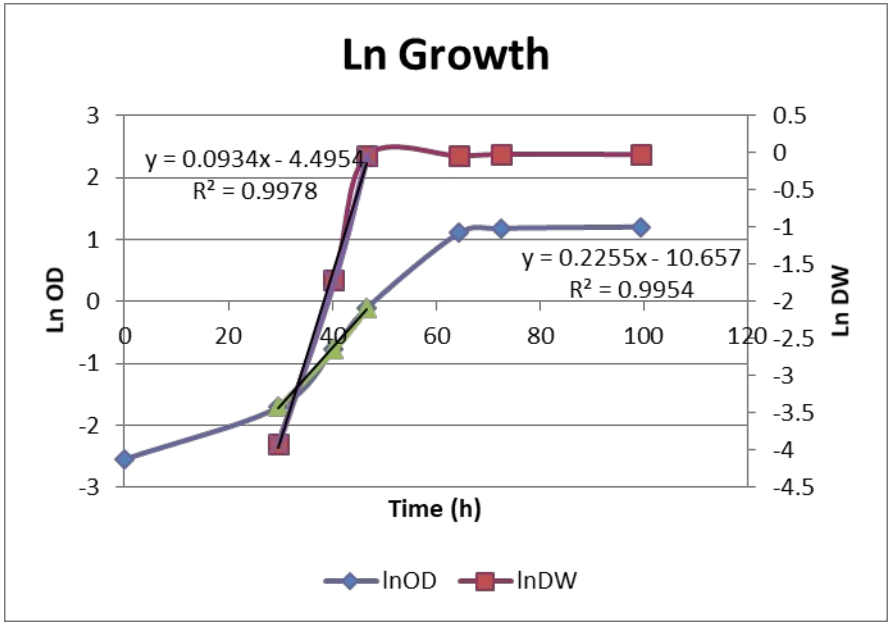

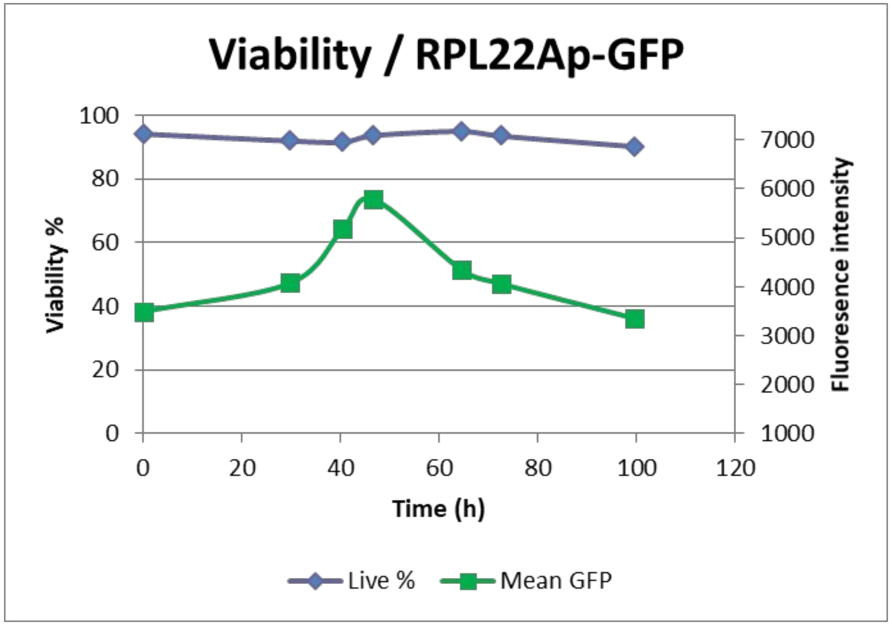

TMBAH58 0x (Alt1Δ::NatMX)

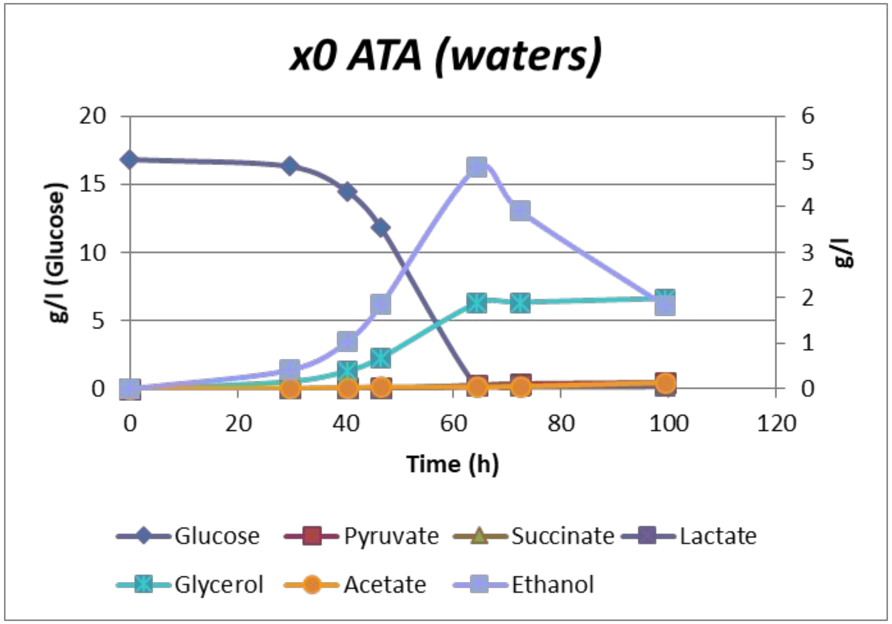

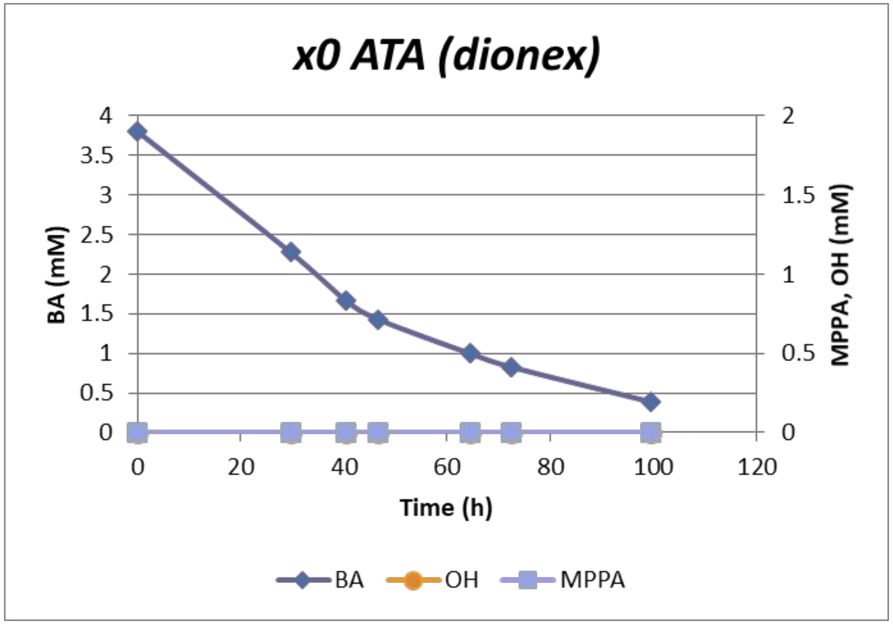

Figure S4
p4

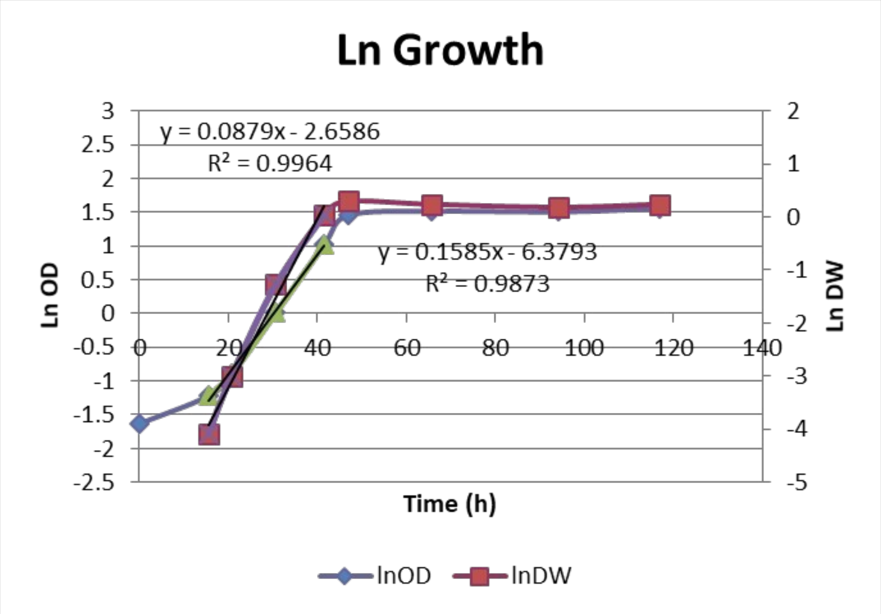

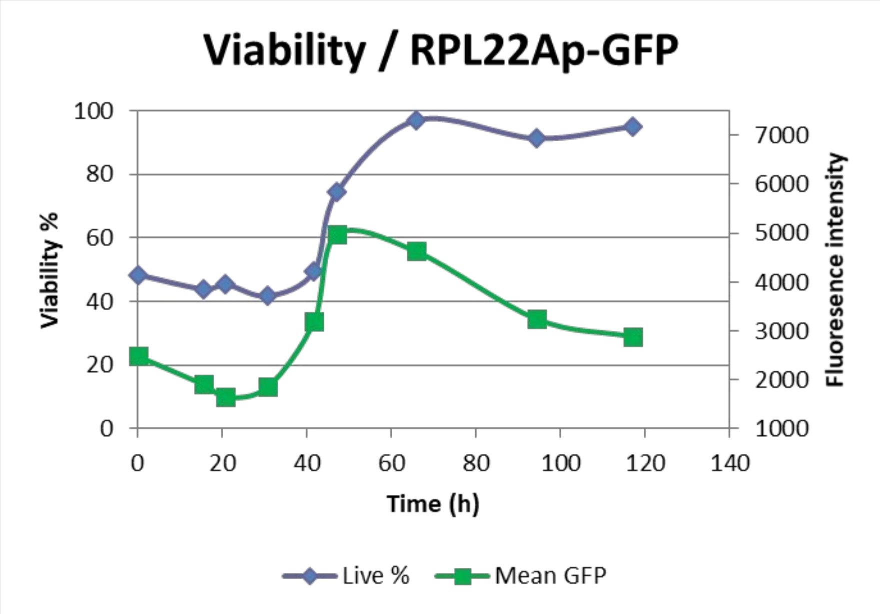

TMBAH61 [A] 1x (Alt1Δ::ATA)

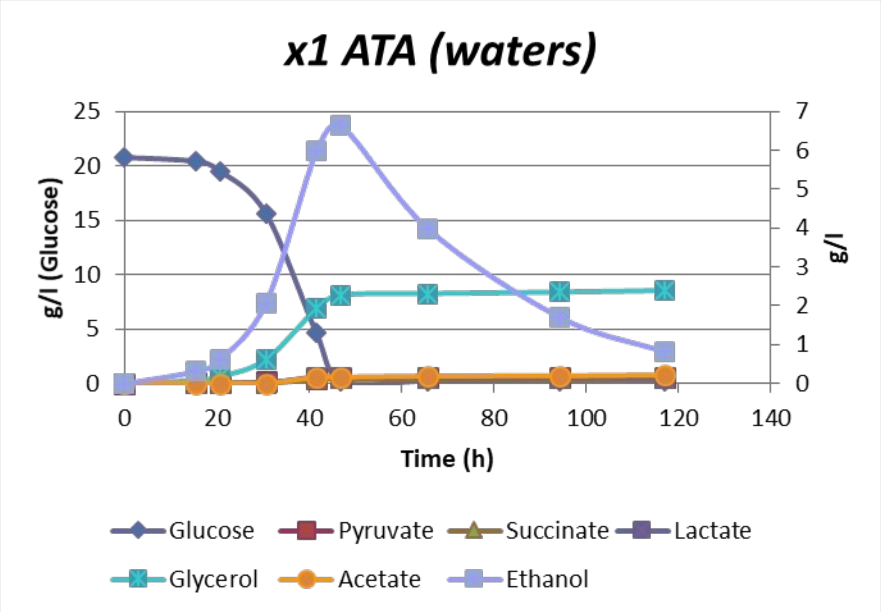

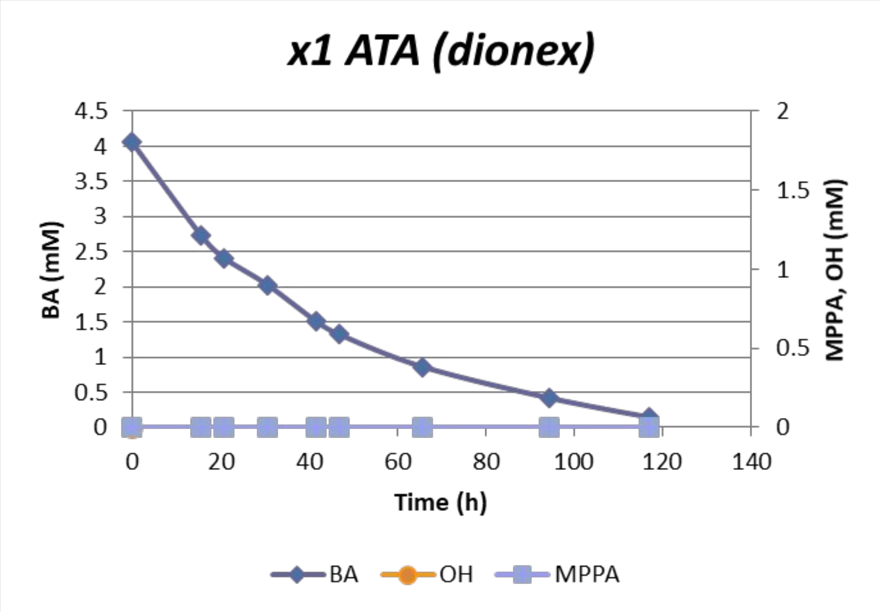

Figure S4
p5

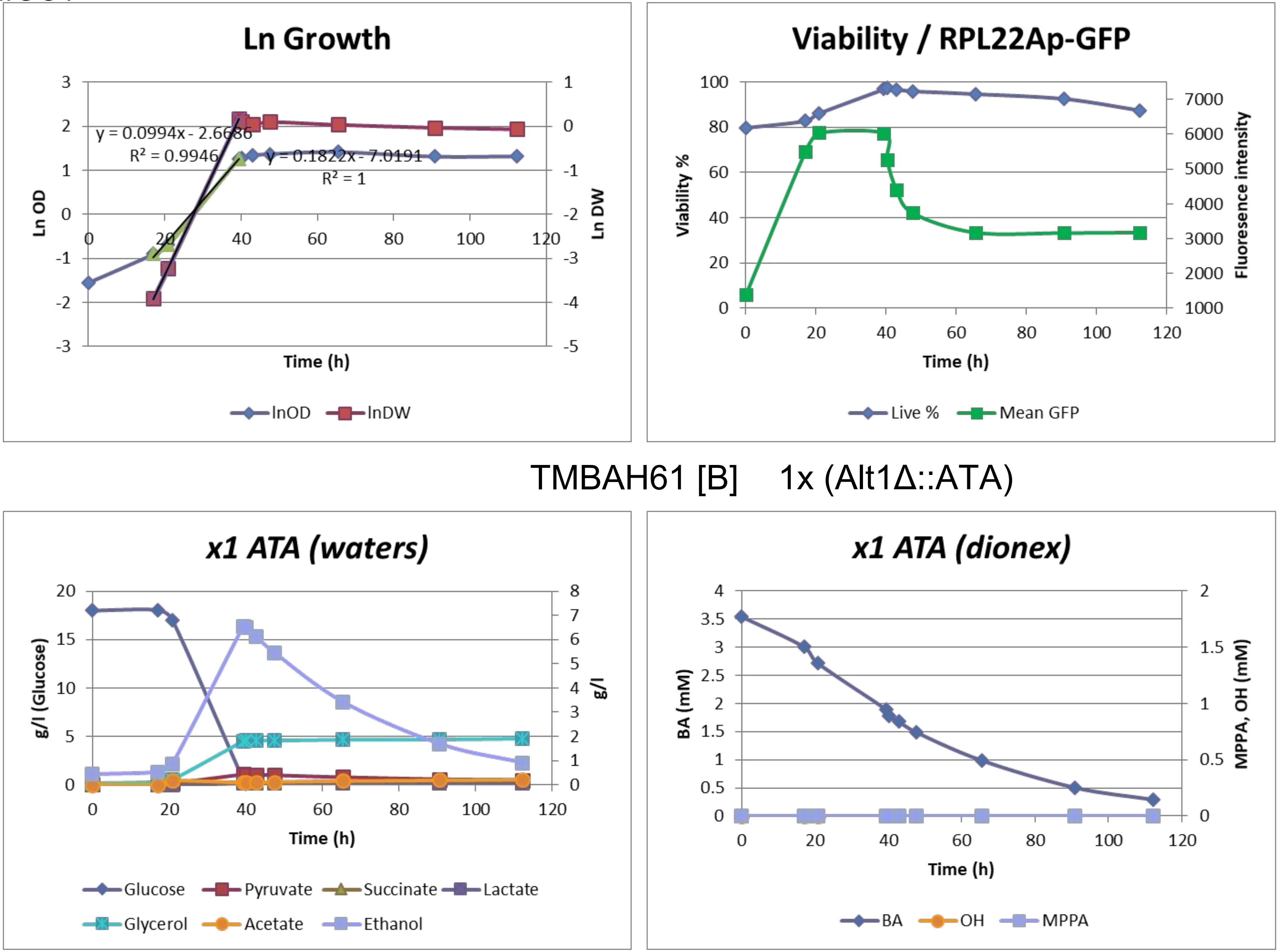

Ln Growth
Ln OD
Ln DW
Time (h)
y = 0.0994x - 2.6686
R² = 0.9946
y = 0.1822x - 7.0191
R² = 1
lnOD
lnDW
Viability / RPL22Ap-GFP
Viability %
Fluoresence intensity
Time (h)
Live %
Mean GFP
TMBAH61 [B] 1x (Alt1Δ::ATA)
x1 ATA (waters)
g/l (Glucose)
g/l
Time (h)
Glucose
Pyruvate
Succinate
Lactate
Glycerol
Acetate
Ethanol
x1 ATA (dionex)
BA (mM)
MPPA, OH (mM)
Time (h)
BA
OH
MPPA

Figure S4
p6

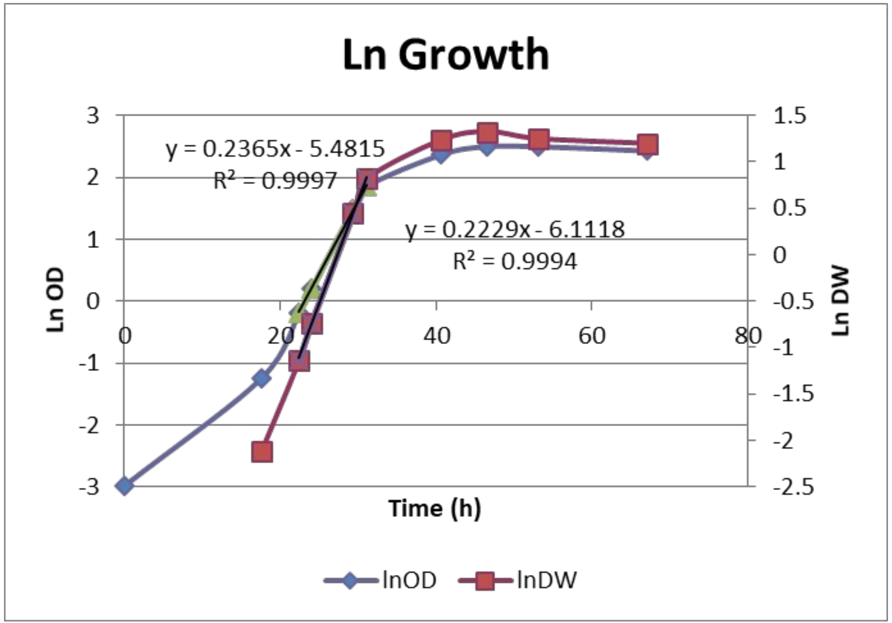

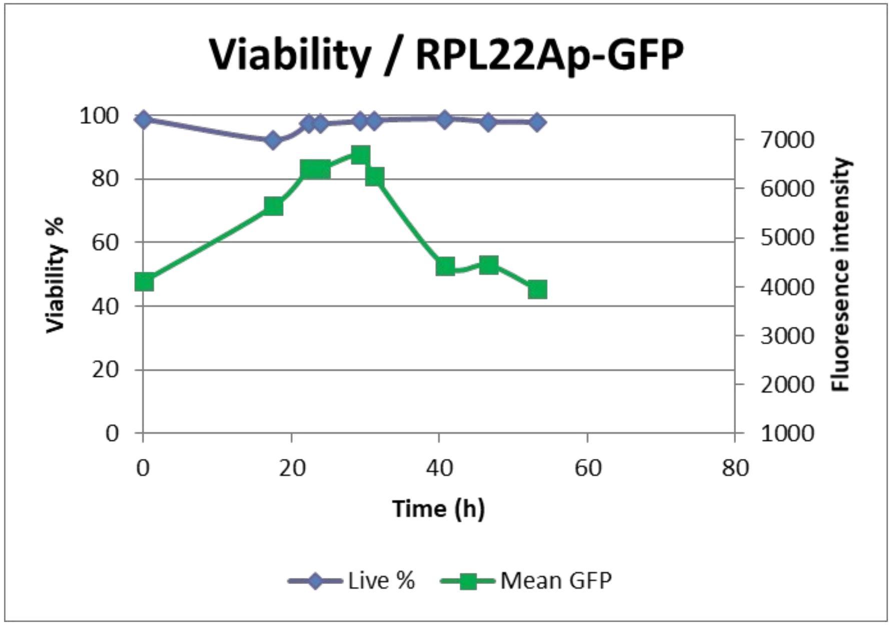

TMBAH60 1x (Alt1Δ::ATA) 113-7D

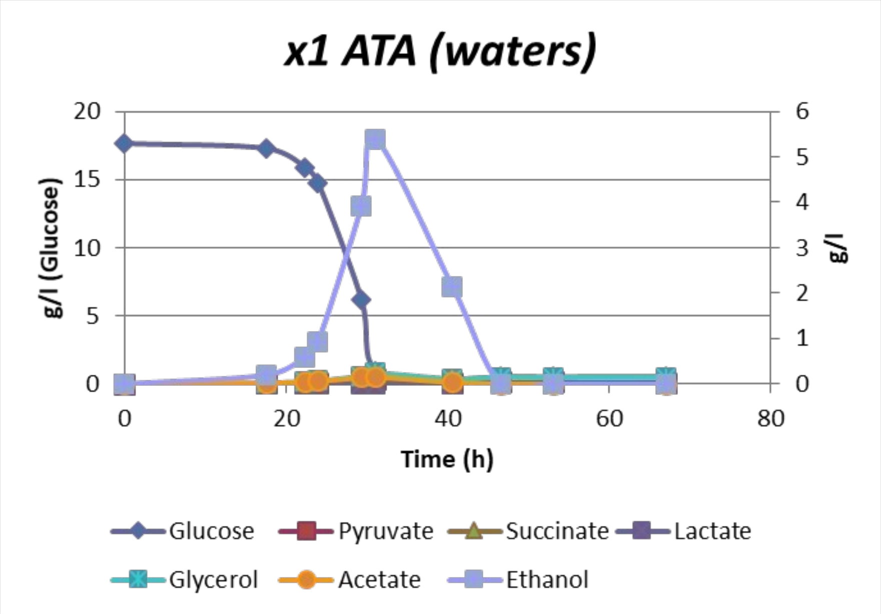

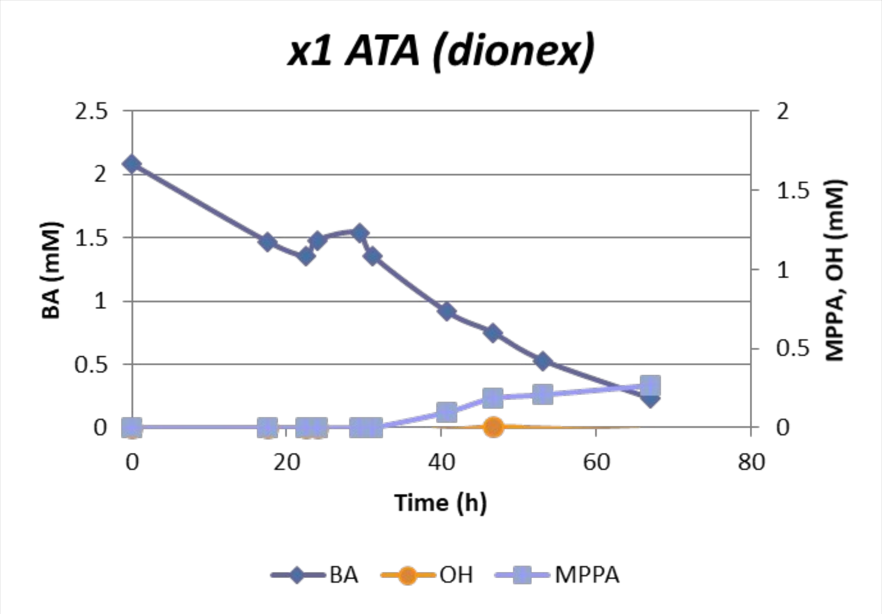

Figure S4
p7

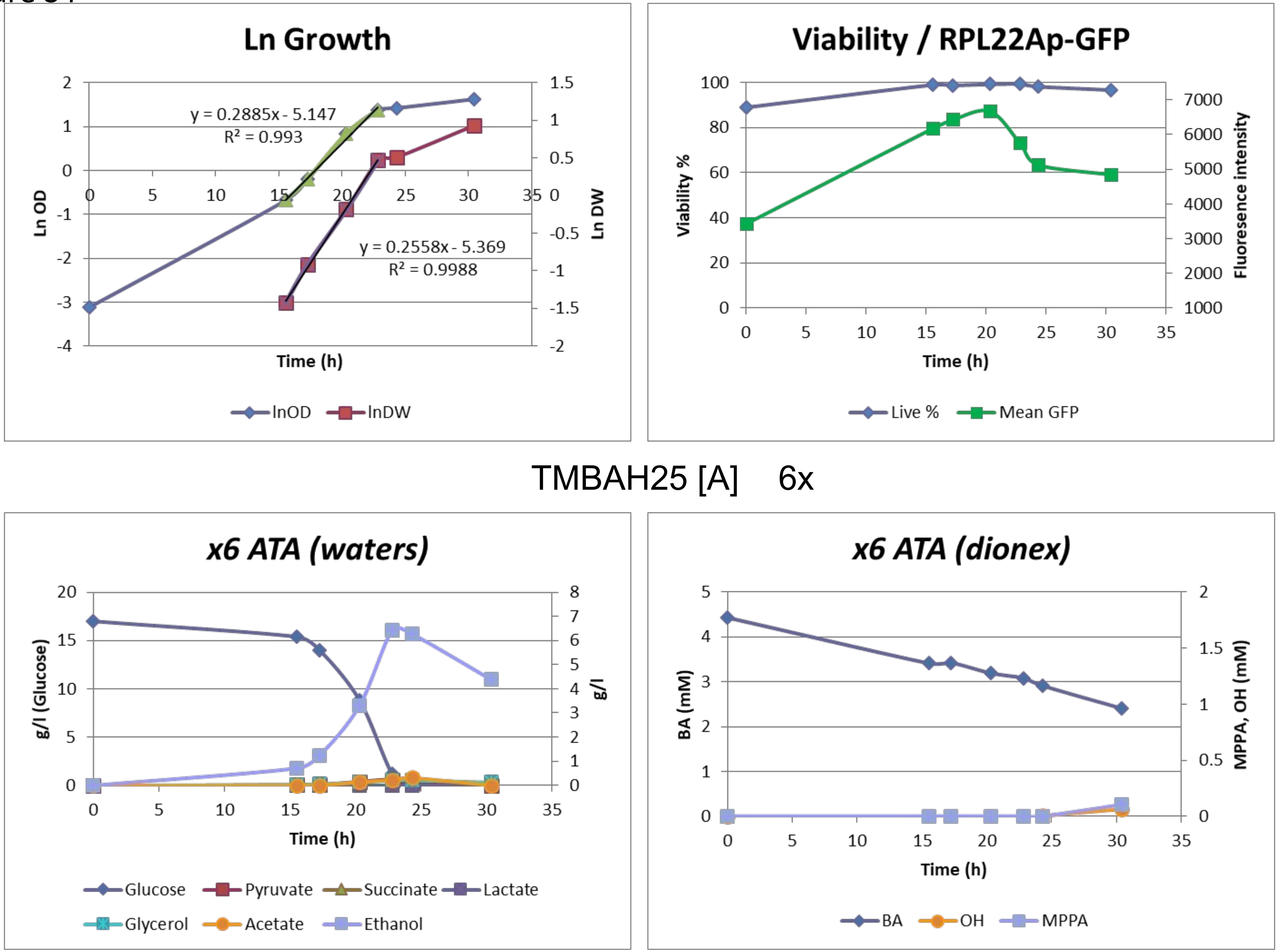
Ln Growth
y = 0.2885x - 5.147
R² = 0.993
y = 0.2558x - 5.369
R² = 0.9988
Ln OD
Ln DW
Time (h)
lnOD
lnDW
Viability / RPL22Ap-GFP
Viability %
Fluoresence intensity
Time (h)
Live %
Mean GFP
TMBAH25 [A] 6x
x6 ATA (waters)
g/l (Glucose)
g/l
Time (h)
Glucose
Pyruvate
Succinate
Lactate
Glycerol
Acetate
Ethanol
x6 ATA (dionex)
BA (mM)
MPPA, OH (mM)
Time (h)
BA
OH
MPPA

Figure S4
p8

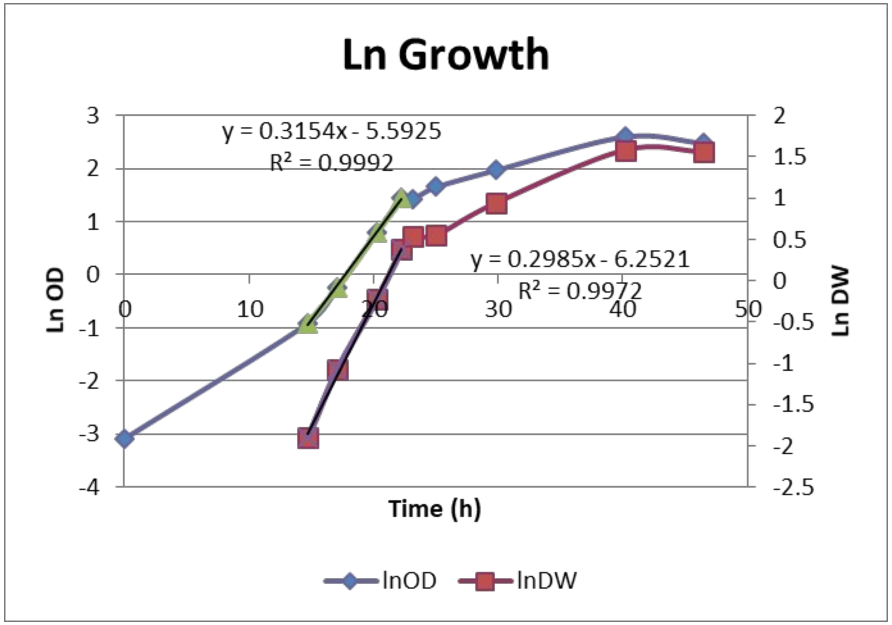

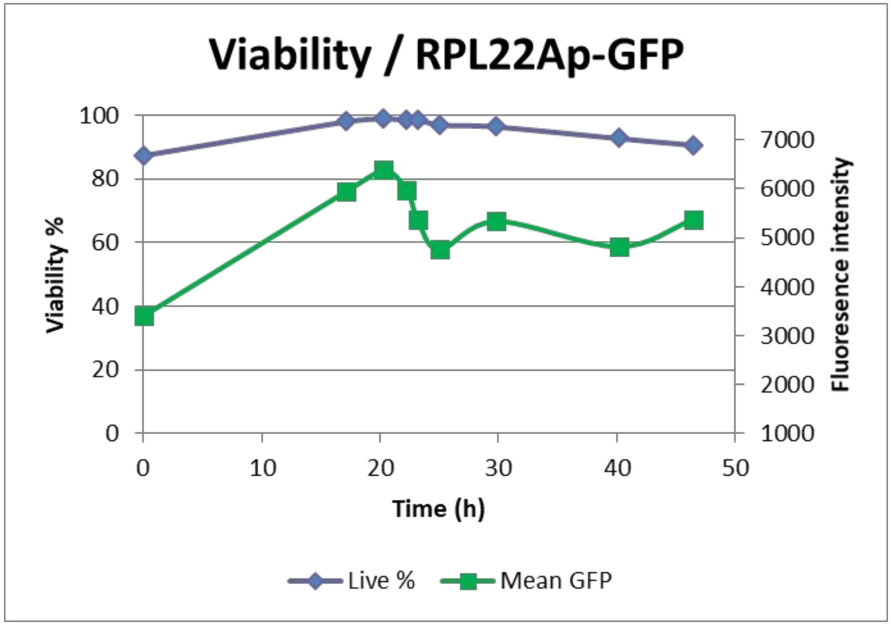

TMBAH25 [B] 6x

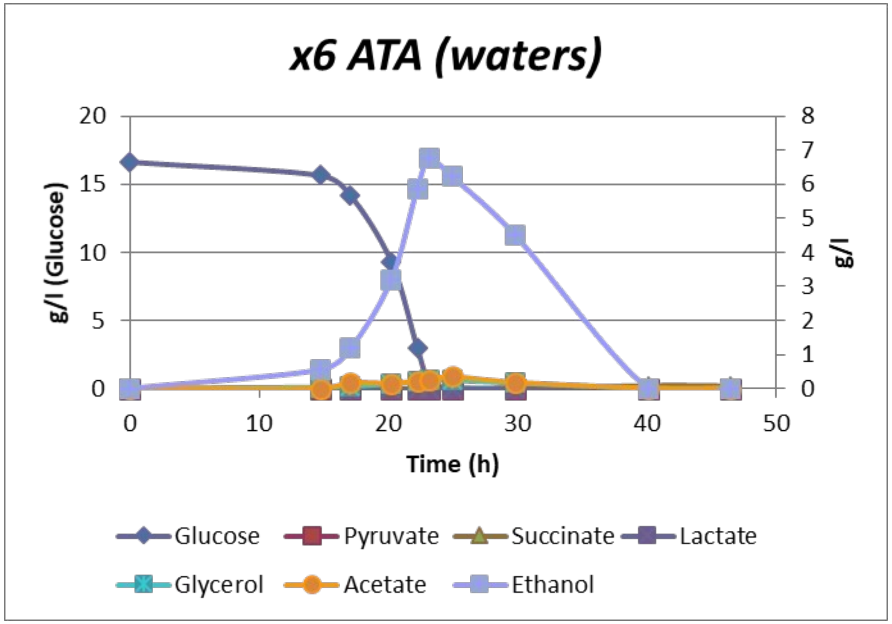

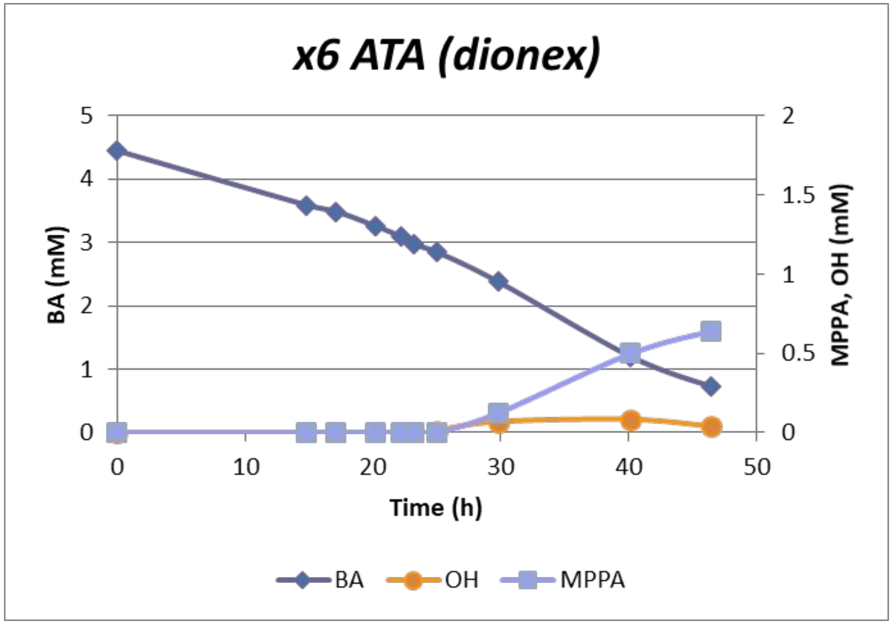

Figure S4
p9

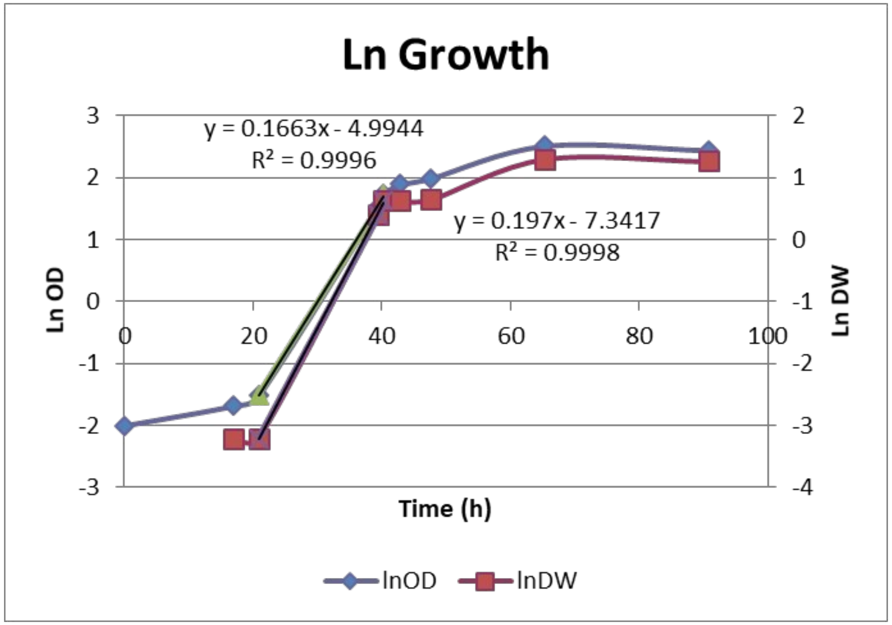

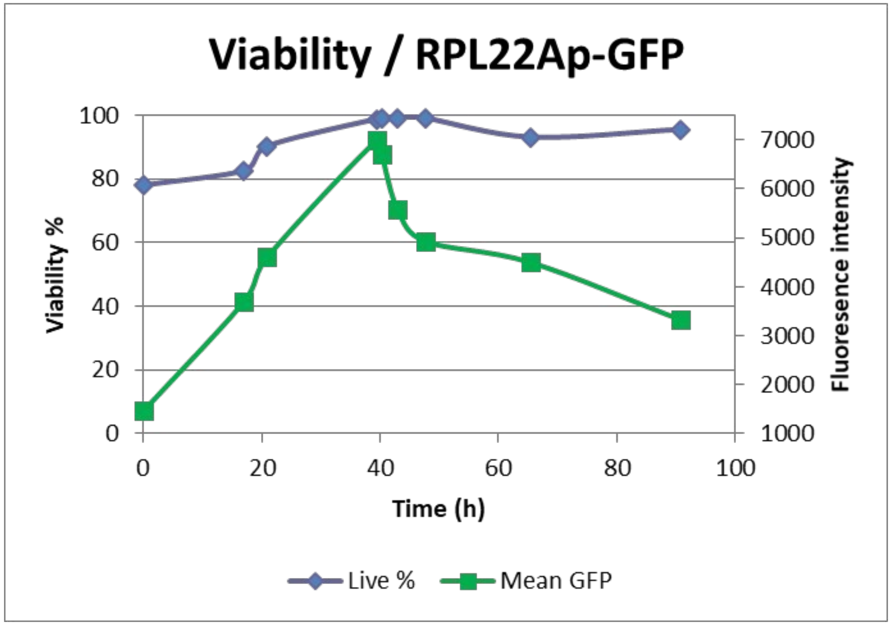

TMBAH62 [A]   7x (Alt1Δ::ATA)

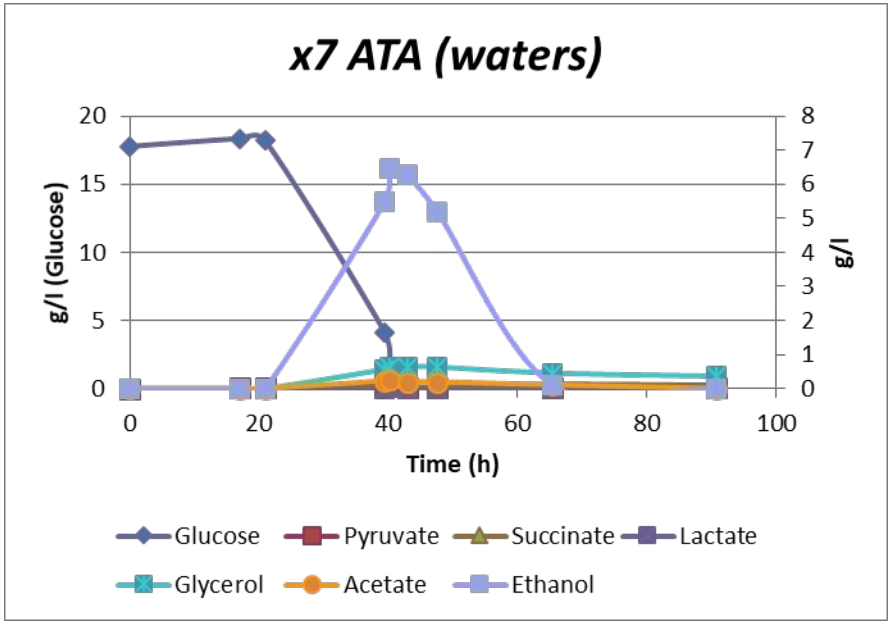

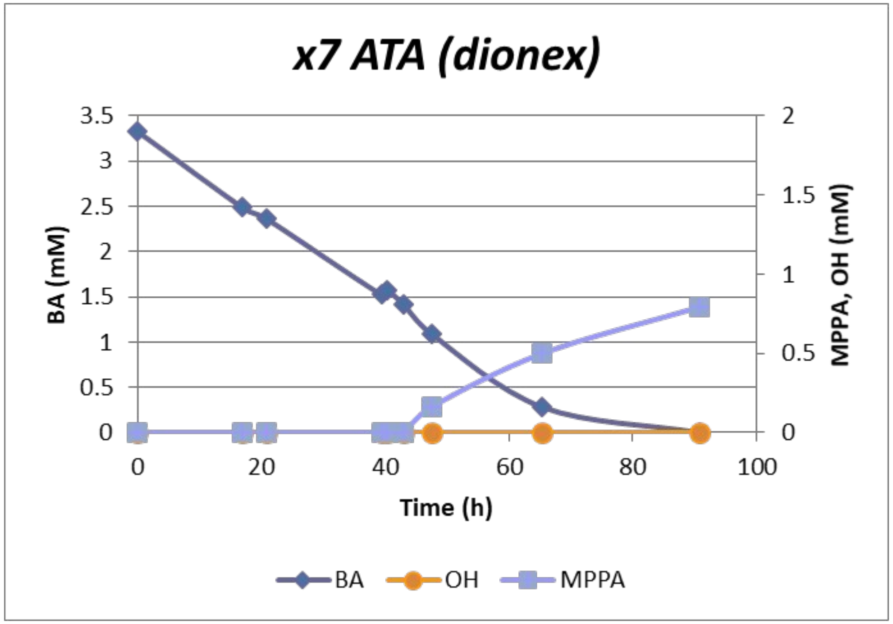

Figure S4

p10

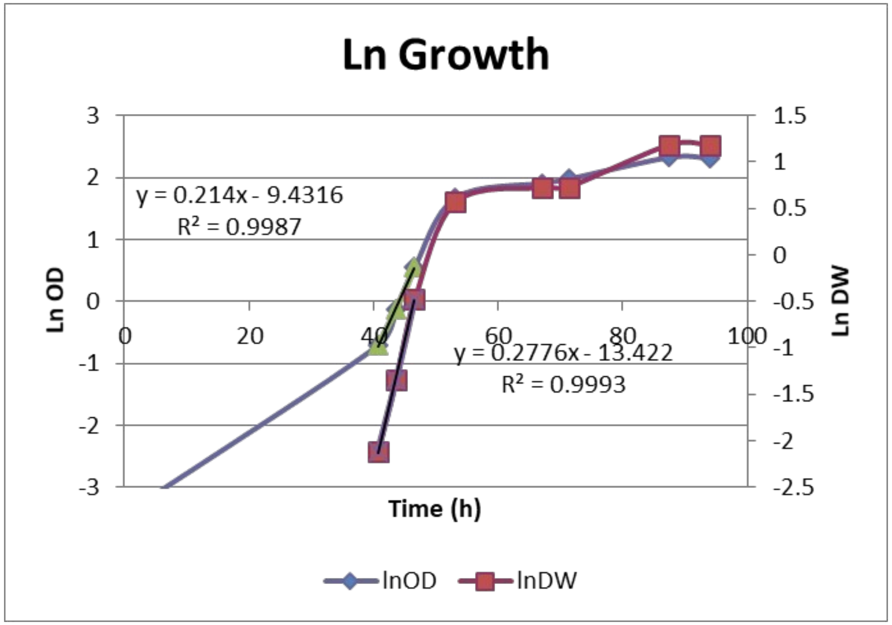

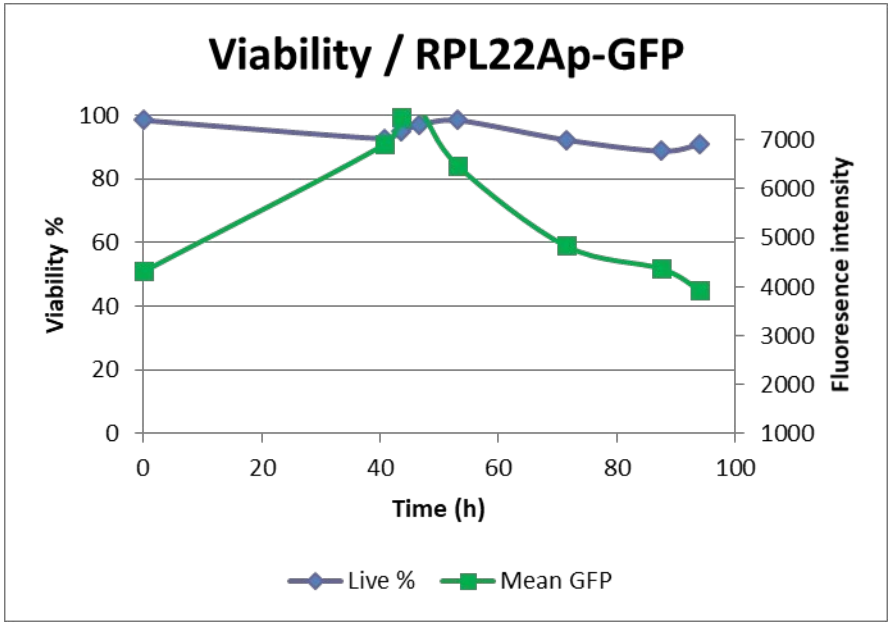

TMBAH62 [B] 7x (Alt1Δ::ATA)

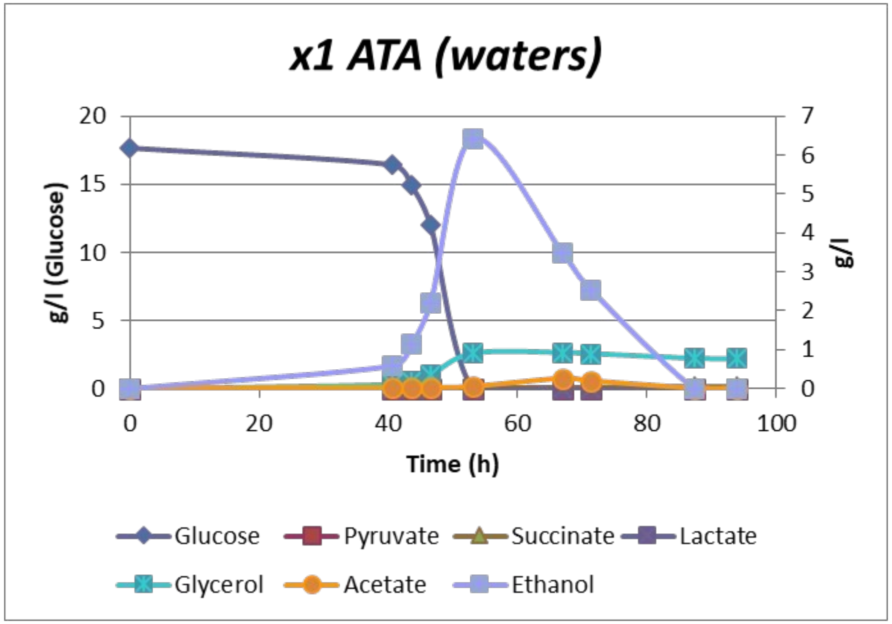

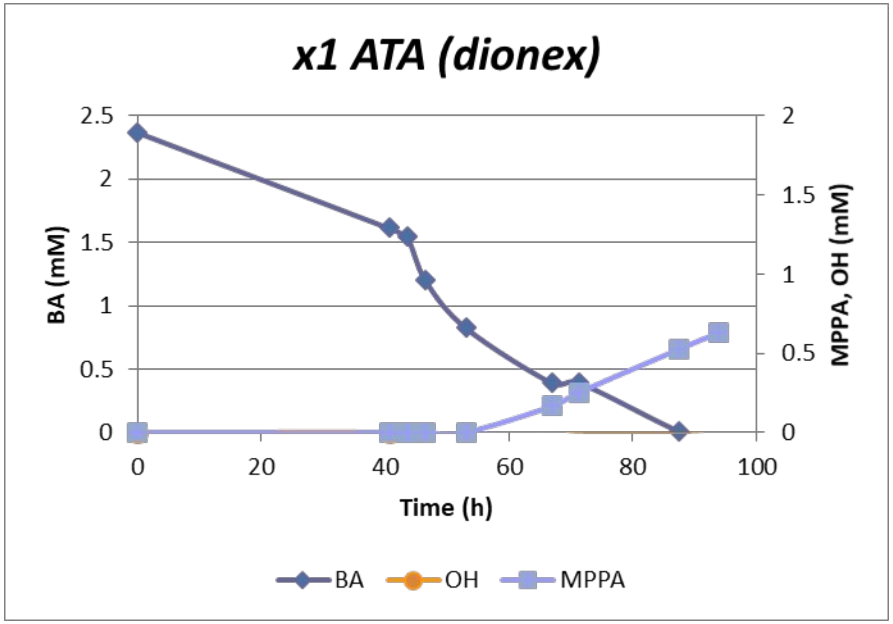

Supplement: Supplementary file 1 — Supplementary Material 1 [file 41598_2025_1182_MOESM1_ESM.pdf]
