# Supplementary material for: Biocatalytic reductive amination with CRISPR-Cas9 engineered yeast
Source: Sci Rep. 2025 May 15;15:16972. doi: 10.1038/s41598-025-01182-0 (PMC12081890; doi:10.1038/s41598-025-01182-0)
Supplement: Supplementary file 3 — Supplementary Material 3 [file 41598_2025_1182_MOESM3_ESM.pdf]

Supplementary information

Whole-cell bio-catalytic reductive amination with CRISPR/Cas9 engineered yeast

Arne Hagman[1,2,], Olof Stenström[3], Göran Carlström[3], Mikael Akke[3], Carl Grey[2], Magnus Carlquist[1]

1. Division of Applied Microbiology, Lund university
2. Division of Biotechnology, Lund university
3. Division of Biophysical Chemistry, Center for Molecular Protein Science, Lund University

Table of contents **Page**

Table of contents	Page
Figure S1. MPPA yield	2
Figure S2. Green fluorescence	3
Figure S3. Detection of endpoint 4-Phenylbutan-2-ol (OH)	4
Figure S4. Characterized strains	See separate file
Figure S5. BA absorption	5
Figure S6. Stoichiometric balance (BA, OH, MPPA)	6
Figure S7. Downscaling to 25ml and 1.5ml (AH61)	7
Figure S8. Downscaling to 25ml and 1.5ml (AH62)	8
Figure S9. Compounds identified by ^{15}N spectra	9
Figure S10. Transaminase activity assay with pure Cv-ATA	10

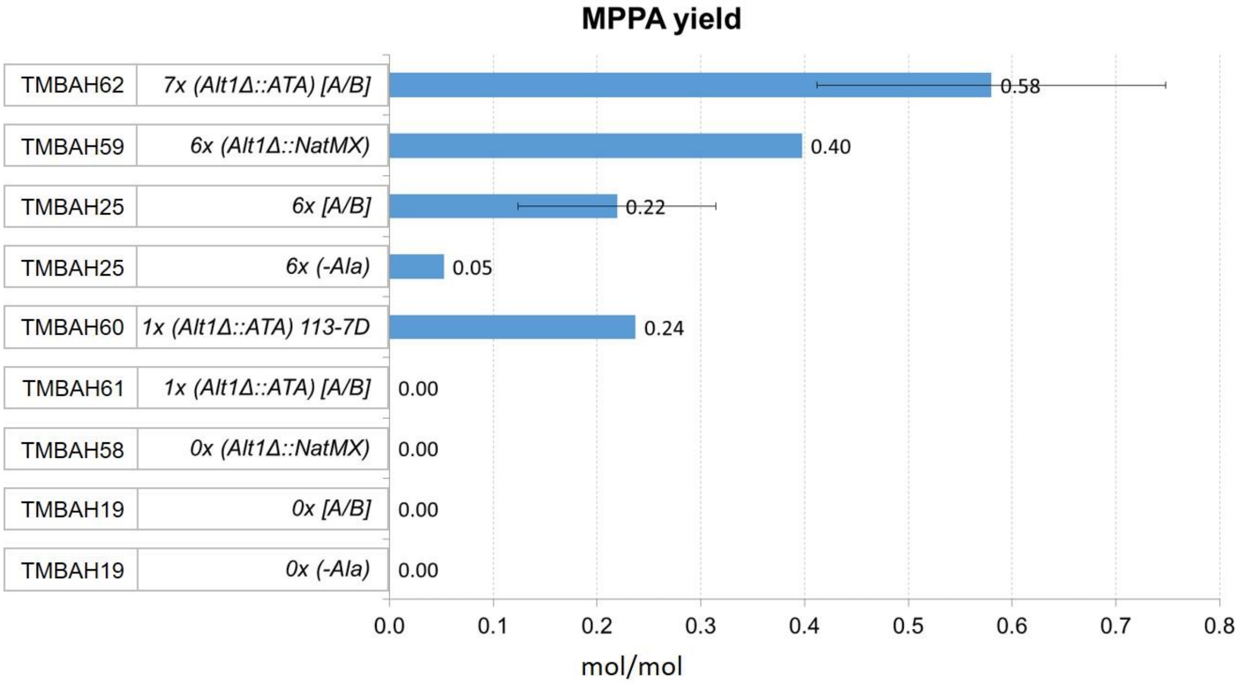

Figure S1. Production strains with six (6x) and seven (7x) copies of ATA, and their references with zero (0x) or one (1x) copy of ATA were tested for MPPA production in the presence of alanine, and without addition of the amine donor (-Ala) in bioreactors. Error bars corresponds to one standard deviation of two biological replicates.

A

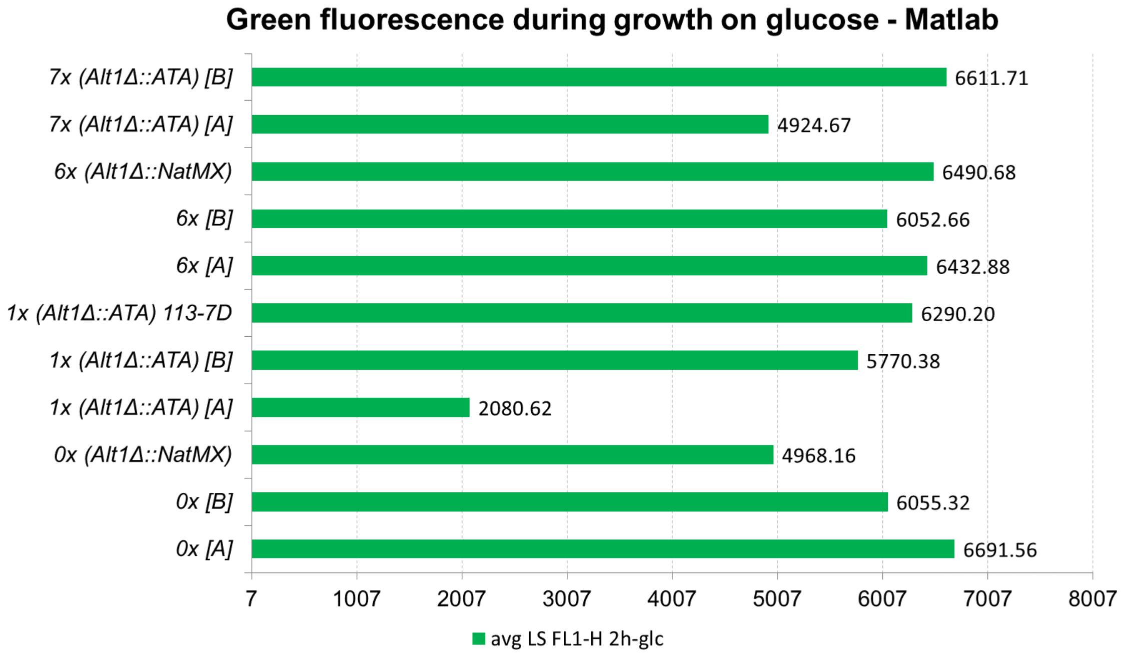

B

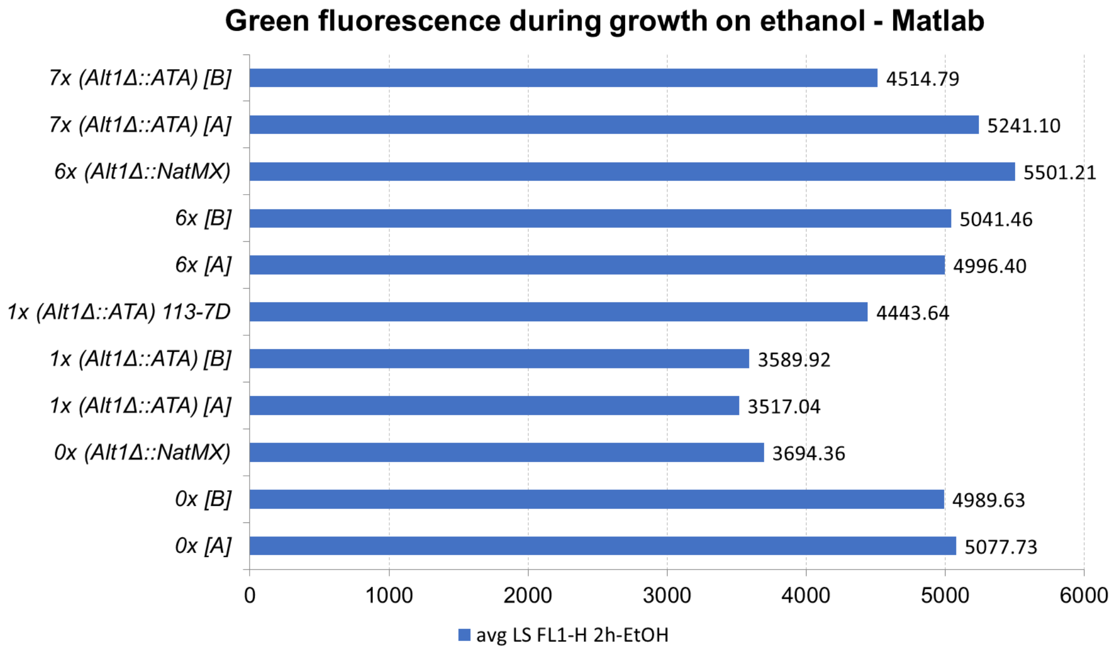

Figure S2. (A) Average green fluorescence for each strain during growth on glucose. (B) Average green fluorescence for each strain during growth on ethanol. Group (II) yeasts (with ALT1 deleted, and up to 1 copy of ATA) have reduced fluorescence as a result of reduced growth on glucose, and growth defect on ethanol.

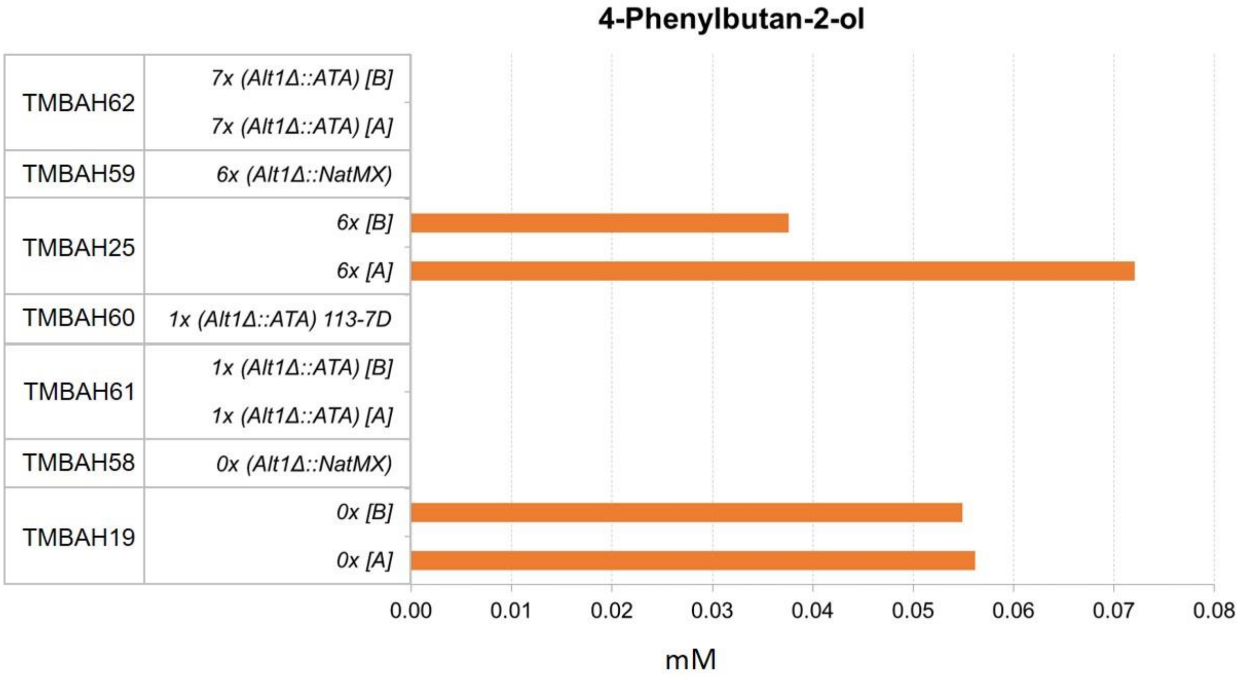

Figure S3. Detection of 4-Phenylbutan-2-ol (OH) at the end of each experiment.

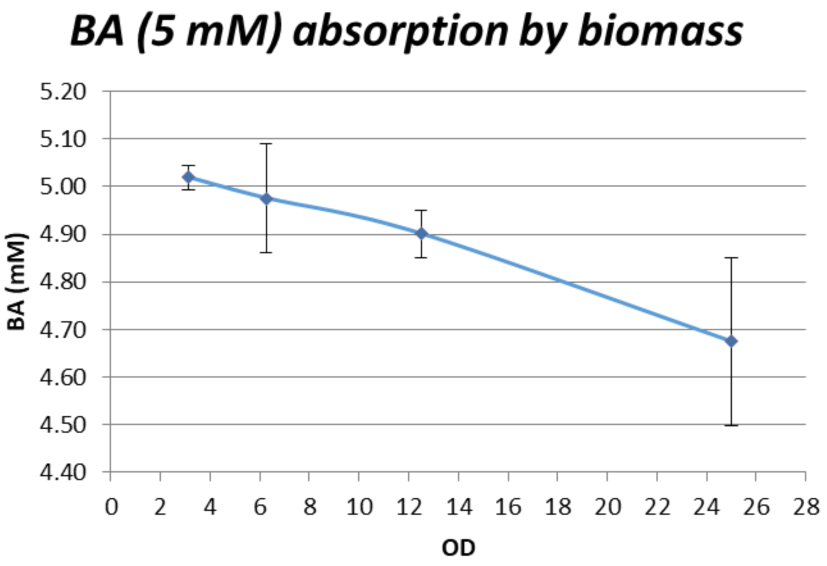

Figure S5. BA concentration as a function of concentration of abiotic cells (OD620nm), demonstrates that a part of the BA binds to abiotic cells. Error bars corresponds to one standard deviation of two biological replicates.

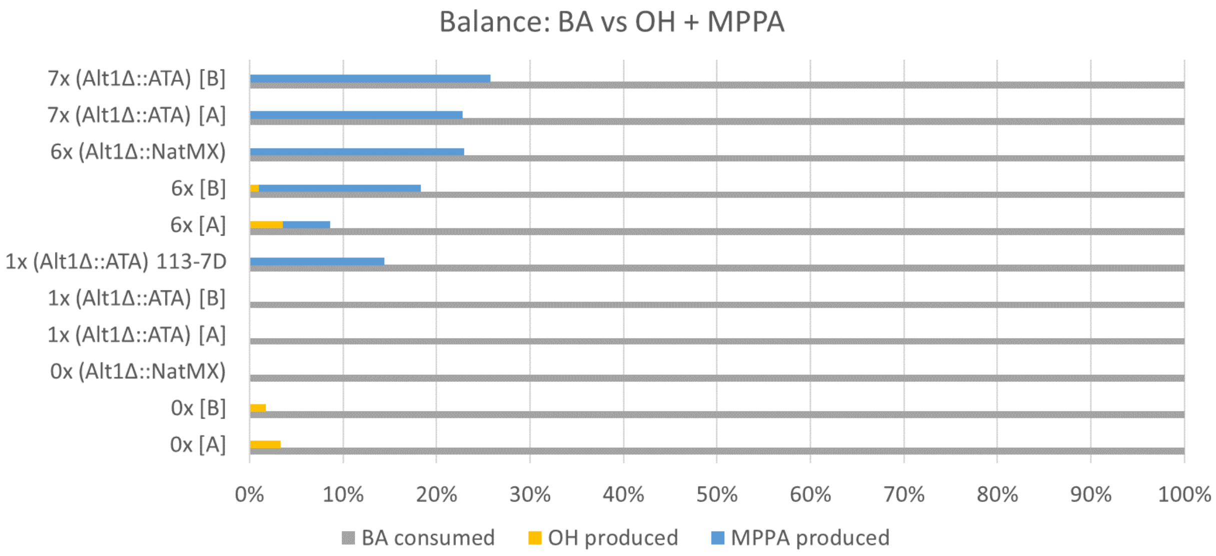

Figure S6. Stoichiometric balance of formed OH and MPPA, and the total amount of consumed BA among all characterized strains.

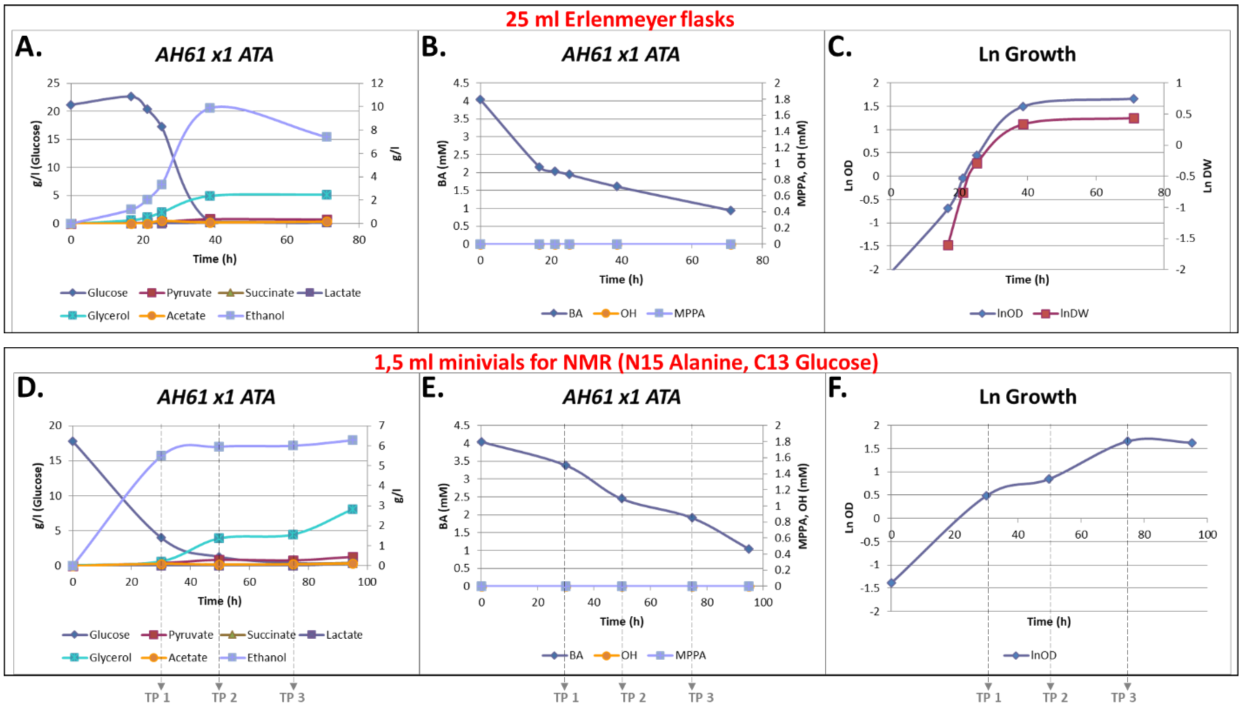

Figure S7. Downscaling experiments from 250ml to 25ml (erlenmeyer) and 1.5ml (mini-vial) for NMR. Reference strain TMBAH61

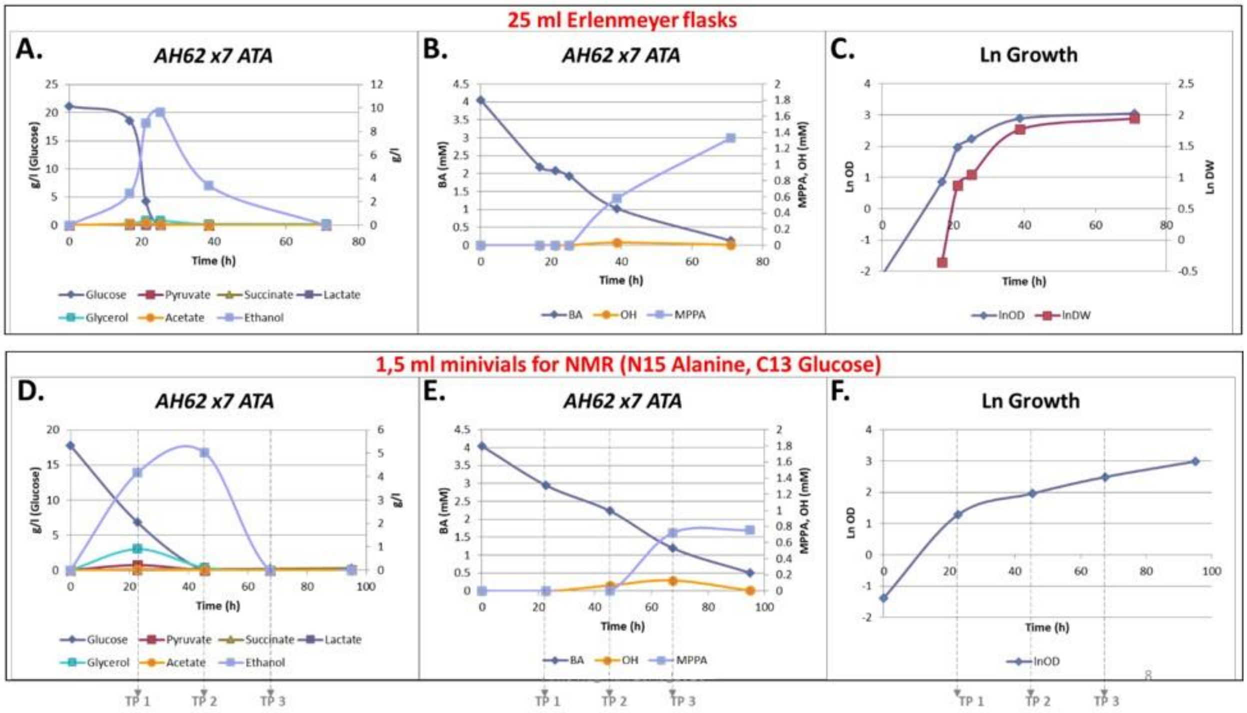

Figure S8. Downscaling experiments from 250ml to 25ml (erlenmeyer) and 1.5ml (mini-vial) for NMR. Production strain TMBAH62

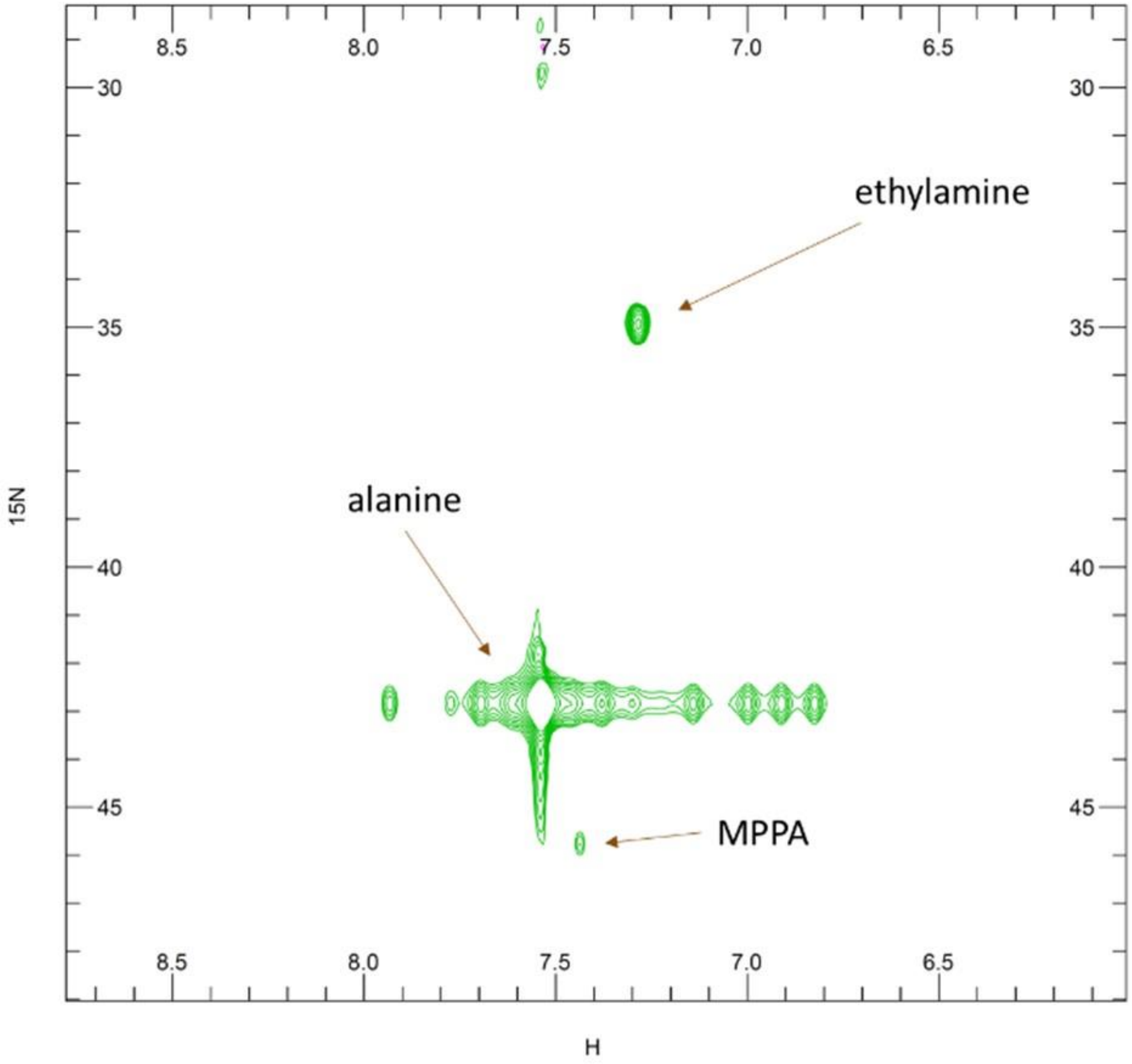

Figure S9. Compounds identified by ^{15}N spectra. Three compounds could be detected and identified by NMR. Alanine and MPPA are previously known products. The third, previously unknown product was identified as ethylamine.

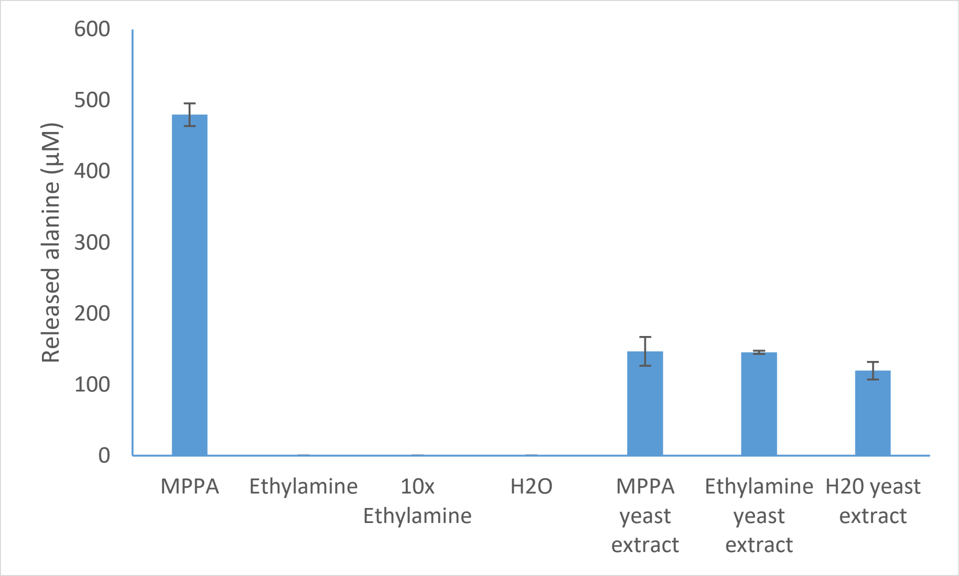

Figure S10. Transaminase activity assay with pure Cv-ATA and cell-free yeast extract/lysate from Cen.PK 113-7d. The reaction was performed at 30 degrees in HEPES-buffer at pH 7, containing 2 mg/L Cv-ATA or yeast lysate, 1 mM PLP, 500 µM pyruvate, and 5 mM MPPA or 5mM, or 50 mM ethylamine. MQ-water was used as negative control instead of amine donors MPPA or ethylamine. The reaction ran for 30 minutes and was quenched by boiling the sample for 10 minutes in a heat block. The concentration of alanine was measured with an Alanine Kit (Cell Biolabs) according to the instructions from the manufacturer.
